# Low-Value Surgical Procedures in Low- and Middle-Income Countries: A Systematic Scoping Review

**DOI:** 10.1001/jamanetworkopen.2023.42215

**Published:** 2023-11-07

**Authors:** Loai Albarqouni, Eman Abukmail, Majdeddin MohammedAli, Sewar Elejla, Mohamed Abuelazm, Hosam Shaikhkhalil, Thanya Pathirana, Sujeewa Palagama, Emmanuel Effa, Eleanor Ochodo, Eulade Rugengamanzi, Yousef AlSabaa, Ale Ingabire, Francis Riwa, Burhan Goraya, Mina Bakhit, Justin Clark, Morteza Arab-Zozani, Suzanna Alves da Silva, C. S. Pramesh, Verna Vanderpuye, Eddy Lang, Deborah Korenstein, Karen Born, Stephen Tabiri, Adesoji Ademuyiwa, Ashraf Nabhan, Ray Moynihan

**Affiliations:** 1 Institute for Evidence-Based Healthcare, Faculty of Health Sciences and Medicine, Bond University, Gold Coast, Australia; 2 Medicine & Health Sciences Faculty, Department of Medicine, An-Najah National University, Nablus, Palestine; 3 Faculty of Medicine, Islamic University of Gaza, Gaza Strip, Palestine; 4 Faculty of Medicine, Tanta University, Tanta, Egypt; 5 School of Medicine and Dentistry, Griffith University, Sunshine Coast, Australia; 6 Department of Internal Medicine, Faculty of Clinical Sciences, College of Medical Sciences, University of Calabar, Calabar, Nigeria; 7 Centre for Global Health Research, Kenya Medical Research Institute, Kismu City, Kenya; 8 Centre for Evidence-Based Health Care, Department of Global Health, Faculty of Medicine and Health Sciences, Stellenbosch University, Stellenbosch, South Africa; 9 Department of Clinical Oncology, Muhimbili University of Health and Allied Sciences, Dar es Salaam, Tanzania; 10 Faculty of Medicine, Al-Azhar University of Gaza, Gaza Strip, Palestine; 11 Princess Margaret Cancer Centre, Toronto, Ontario, Canada; 12 Social Determinants of Health Research Center, Birjand University of Medical Sciences, Birjand, Iran; 13 Department of Epidemiology, HCor Hospital, São Paulo, Brazil; 14 Tata Memorial Centre, Homi Bhabha National Institute, Mumbai, India; 15 National Centre for Radiotherapy, Oncology and Nuclear Medicine, Korle Bu Teaching Hospital, Accra, Ghana; 16 Department of Emergency Medicine, Cumming School of Medicine, University of Calgary, Calgary, Alberta, Canada; 17 Department of Medicine, Icahn School of Medicine at Mount Sinai, New York, New York; 18 Institute of Health Policy, Management and Evaluation, Dalla Lana School of Public Health, Faculty of Medicine, University of Toronto, Toronto, Ontario, Canada; 19 Department of Surgery, University for Development Studies–School of Medicine and Tamale Teaching Hospital, Tamale, Ghana; 20 Paediatric Surgery Unit, Department of Surgery, Faculty of Clinical Sciences, College of Medicine of the University of Lagos and Lagos University Teaching Hospital, Idi Araba, Lagos; 21 Department of Obstetrics and Gynecology, Faculty of Medicine, Ain Shams University, Cairo, Egypt

## Abstract

**Importance:**

Overuse of surgical procedures is increasing around the world and harms both individuals and health care systems by using resources that could otherwise be allocated to addressing the underuse of effective health care interventions. In low- and middle-income countries (LMICs), there is some limited country-specific evidence showing that overuse of surgical procedures is increasing, at least for certain procedures.

**Objectives:**

To assess factors associated with, extent and consequences of, and potential solutions for low-value surgical procedures in LMICs.

**Evidence Review:**

We searched 4 electronic databases (PubMed, Embase, PsycINFO, and Global Index Medicus) for studies published from database inception until April 27, 2022, with no restrictions on date or language. A combination of MeSH terms and free-text words about the overuse of surgical procedures was used. Studies examining the problem of overuse of surgical procedures in LMICs were included and categorized by major focus: the extent of overuse, associated factors, consequences, and solutions.

**Findings:**

Of 4276 unique records identified, 133 studies across 63 countries were included, reporting on more than 9.1 million surgical procedures (median per study, 894 [IQR, 97-4259]) and with more than 11.4 million participants (median per study, 989 [IQR, 257-6857]). Fourteen studies (10.5%) were multinational. Of the 119 studies (89.5%) originating from single countries, 69 (58.0%) were from upper-middle–income countries and 30 (25.2%) were from East Asia and the Pacific. Of the 42 studies (31.6%) reporting extent of overuse of surgical procedures, most (36 [85.7%]) reported on unnecessary cesarean delivery, with estimated rates in LMICs ranging from 12% to 81%. Evidence on other surgical procedures was limited and included abdominal and percutaneous cardiovascular surgical procedures. Consequences of low-value surgical procedures included harms and costs, such as an estimated US $3.29 billion annual cost of unnecessary cesarean deliveries in China. Associated factors included private financing, and solutions included social media campaigns and multifaceted interventions such as audits, feedback, and reminders.

**Conclusions and Relevance:**

This systematic review found growing evidence of overuse of surgical procedures in LMICs, which may generate significant harm and waste of limited resources; the majority of studies reporting overuse were about unnecessary cesarean delivery. Therefore, a better understanding of the problems in other surgical procedures and a robust evaluation of solutions are needed.

## Key Points

**Question:**

Are unnecessary surgical procedures performed in low- and middle-income countries (LMICs), and what are the associated factors, consequences, and solutions to reduce overuse?

**Findings:**

In this systematic scoping review of 133 studies with more than 9.1 million surgical procedures and 11.4 million participants in 63 LMICs, cesarean delivery was the most commonly examined unnecessary surgery, with estimated rates ranging from 12% to 81%. Consequences of unnecessary surgical procedures included patient harms and costs, and associated factors included private financing.

**Meaning:**

This study found growing evidence of overuse of unnecessary surgical procedures, especially unnecessary cesarean deliveries, in LMICs.

## Teaser

This systematic review investigates the factors associated with, extent and consequences of, and potential solutions for the overuse of low-value or unnecessary surgical procedures in low- and middle-income countries.

## Introduction

Overuse of surgical procedures poses harm to both individuals and health care systems by using or diverting resources that could be applied to address the underuse of effective health care interventions.^1,2,3,4,5,6^ Overuse of surgical procedures refers to low-value surgical procedures that provide little or no benefit and do not outweigh the associated harms to individuals through adverse events, the burden of unnecessary interventions, the increase in health care spending, and the psychosocial impacts of labeling.^1,2,3^ The problem of overuse also threatens the sustainability of health systems by consuming resources that could be allocated to addressing underuse^4^ and underdiagnosis, thereby indirectly causing harm to people with unmet needs.^2^ The problem of overuse of unnecessary services holds greater significance in low- and middle-income countries (LMICs) due to the limited availability of resources.

There is increasing global recognition of the problem of overuse of surgical and other invasive procedures. There is some limited, country-specific evidence showing that overuse of surgical procedures is potentially increasing, at least for certain procedures, in LMICs. For instance, there has been a steady increase in the rates of cesarean delivery (CD),^7^ with considerable variations between and within countries including India, Tanzania, Bangladesh, Turkey, China, and Nepal.^8,9,10,11^ A World Health Organization (WHO)–supported study that plans to enroll around 800 000 women is aiming to reduce unnecessary CDs across 4 LMICs.^12^ Although the extent of the overuse of surgical procedures in LMICs is unknown, these challenges are especially important in LMICs, where health expenditure in relation to the gross domestic product is significantly lower and such waste threatens both population health and the viability of public budgets.^1^ Addressing the problem of overuse of low-value surgical procedures could potentially reduce harm and prevent waste and may assist in supporting efforts to achieve sustainability, fairness, and equity of health systems worldwide, including universal health coverage as a central part of the United Nations Sustainable Development Goals.^1,13^

The problem of overuse of surgical procedures in LMICs and wider related problems of low-value care and overdiagnosis are attracting increasing attention.^14,15,16,17,18,19^ Choosing Wisely, a clinician-led campaign aiming to reduce unnecessary tests, treatments, and procedures, has a far-reaching international impact.^20^ Several LMICs, including Brazil, India, Iran, and some sub-Saharan African countries, are adapting and implementing the campaign.^20,21,22^ Country-specific scoping reviews of the evidence are emerging,^23,24,25,26^ and workshops at the 2019 and 2022 International Preventing Overdiagnosis conferences called for more research and actions addressing overuse of surgical procedures in LMICs, including a new global network.^27,28^ A WHO official has said that as the world moves toward universal health coverage, it is critical to address “the waste and the inadvertent iatrogenic harm” caused by the wider problems of overuse and overdiagnosis.^29^ Against the backdrop of the COVID-19 pandemic, there are increasing calls for health systems to address the harm and waste of unneeded care in postpandemic recovery.^30^

To identify gaps in knowledge, inform future agendas for research and action, and foster a global collaboration to advance this work, some of us have undertaken a series of scoping reviews of the available evidence on overdiagnosis^31^ and overuse of medications^32^ in LMICs. In this third scoping review, we aimed to review available evidence about the factors associated with and the extent, consequences, and solutions of the overuse of surgical procedures in LMICs.

## Methods

### Protocol and Registration

We conducted this systematic scoping review according to the Joanna Briggs Institute (JBI) guidance.^33^ This study followed the Preferred Reporting Items for Systematic Reviews and Meta-analyses (PRISMA) Extension for Scoping Reviews guideline.^34^

### Eligibility Criteria

We used broad inclusion criteria to identify studies reporting on the overuse of surgical procedures in LMICs. With use of the JBI’s Population, Concept, Context framework, the population was patients undergoing surgery, the concept was the overuse of surgical procedures, and the context was LMICs. Studies were deemed eligible for inclusion if they met the criteria in the following subsections.

#### Publication Type and Study Design

We included articles published in peer-reviewed scientific journals as well as gray literature (eg, a government report). We included secondary (eg, systematic reviews) and primary (eg, interventional [randomized clinical trials] and observational [cohort, case-control, and cross-sectional]) studies. We also included qualitative studies. We excluded case reports and case series, nonresearch opinion pieces (eg, editorials, analysis, and commentaries), literature reviews, modeling studies, and protocols of primary and secondary studies.

#### Concepts—Study Types or Topics

For this broad, scoping review, we accepted the definition of *overuse* used by each study’s authors. A surgical procedure was defined as any procedure performed with the goal of correcting deformities or defects, of repairing injuries, or for the cure of certain diseases, as specified by the National Center for Biotechnology Information.^35^ We included studies with a major focus or objective related to any of the following 4 themes: extent, consequences, associated factors, and solutions. Extent included investigation or estimation of the extent of the overuse of surgical procedures irrespective of the type of surgery (eg, open or minimally invasive, including open, minimally invasive, laparoscopic, and endoscopic procedures). Consequences consisted of evaluation of the impact of overuse of surgical procedures (eg, physical and psychological harms of overuse, financial and human resource use, and equity on individual and community levels). Associated factors included investigation of the factors associated with the overuse of surgical procedures, and solutions consisted of evaluation of the effects of interventions to reduce the overuse of surgical procedures (eg, interventions to reduce unnecessary CDs).

We excluded studies in which the overuse of surgical procedures was not a main focus or finding of the study (eg, studies that investigated inappropriate endoscopies in which the main focus was on the diagnostic procedure rather than the therapeutic surgical procedure). We also excluded comparative studies evaluating the effectiveness of different modalities of surgical procedures (eg, open vs laparoscopic surgery).

#### Context—Study Locations

We included studies conducted in 1 or more LMICs (ie, low-income, lower-middle–income, and upper-middle–income countries, as defined by the World Bank in 2021) (eAppendix 1 in Supplement 1).^36^ Studies including LMICs and non-LMICs were included if the majority of the data were from LMICs or they reported a subgroup analysis of the data pertaining to LMICs.

### Information Sources and Search Strategy

#### Electronic Databases

We searched 4 electronic databases (PubMed, Embase, PsycINFO, and Global Index Medicus) for articles published from database inception until April 27, 2022. We did not apply any restrictions on language or publication date. We designed a search strategy in PubMed that included a combination of MeSH terms and free-text words related to the following general concept: overuse of surgical procedures or operations. We used the Cochrane Effective Practice and Organisation of Care LMIC search filter to help identify studies relevant to LMICs. Each search string has been translated for use in other databases using the Polyglot Search Translator. The complete search strings for all databases are provided in eAppendix 2 in Supplement 1.

### Study Selection and Screening

Members of our team (L.A., E.A., M.M., SE, M.A., H.S., T.P., S.P., E.E., E.O., E.R., Y.A., A.I., F.R., B.G., M.B., and M.A.Z.) worked in pairs to independently screen the titles and abstracts and then the full text once obtained. Any disagreements were resolved by discussion or by reference to a third member of our team (L.A.). To ensure reliability among screeners, all pairs independently screened a random sample of 20 to 40 citations and continued discussion until acceptable agreement on inclusion and exclusion criteria was attained.

### Data Charting and Extraction

A data charting form was developed and independently piloted on a random sample of 10% of included articles and modified as required based on feedback from within the team. The team members who screened the articles charted and extracted the data and verified a small proportion (5%).

We charted and extracted data on publication and study characteristics, including first author; year; article title; journal of publication; country of the corresponding author; funding sources; study design, duration, and location (country, single or multiple countries); study type (eg, review, primary study); and type of services (eg, primary, community, or secondary level). We also charted and extracted data on concept characteristics, potentially including conditions and surgical procedures studied (eg, name of the surgical procedure, its degree of risk [eg, major or minor], its purpose [eg, elective, urgent, emergent, reconstructive, cosmetic], and its anatomical location), the study’s main theme (presence or estimates of extent, associated factors, consequences, and solutions), and key findings related to the overuse of medications.

### Data Analysis

We categorized included studies by major focus among the main themes (extent, associated factors, consequences, and solutions), surgical procedure (with a specific highlight on surgical procedures that are known to be of low value [eg, CDs or spine and knee surgeries]^37^), and income level of the country or countries. When possible, we summarized the study designs, types of surgical procedures, and key findings. Data were analyzed using R, version 4.0.2 (R Project for Statistical Computing).

## Results

**Figure 1.**
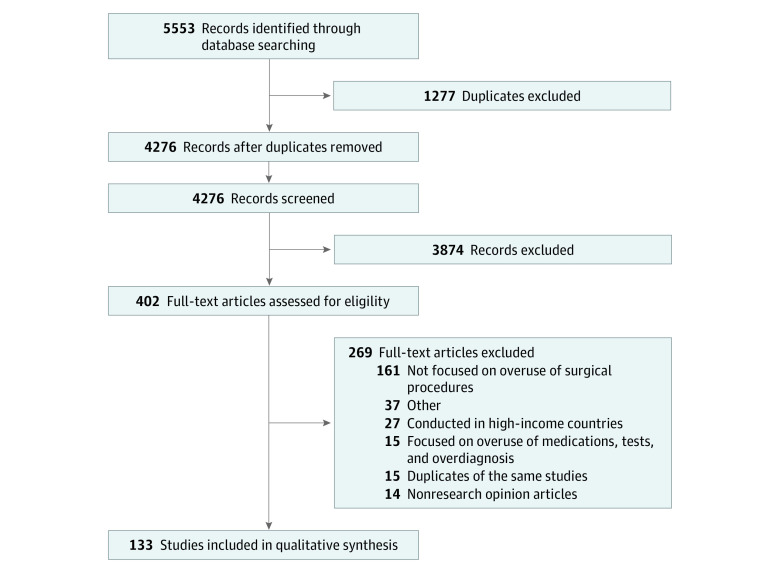
PRISMA Flowchart of the Study Selection Process

A total of 4276 articles were identified. Of those, 402 underwent full-text screening, 269 were excluded, and 133 were included in this review (Figure 1).^9,11,16,17,20,38,39,40,41,42,43,44,45,46,47,48,49,50,51,52,53,54,55,56,57,58,59,60,61,62,63,64,65,66,67,68,69,70,71,72,73,74,75,76,77,78,79,80,81,82,83,84,85,86,87,88,89,90,91,92,93,94,95,96,97,98,99,100,101,102,103,104,105,106,107,108,109,110,111,112,113,114,115,116,117,118,119,120,121,122,123,124,125,126,127,128,129,130,131,132,133,134,135,136,137,138,139,140,141,142,143,144,145,146,147,148,149,150,151,152,153,154,155,156,157,158,159,160,161,162,163,164,165^

### Characteristics of Included Studies

**Figure 2.**
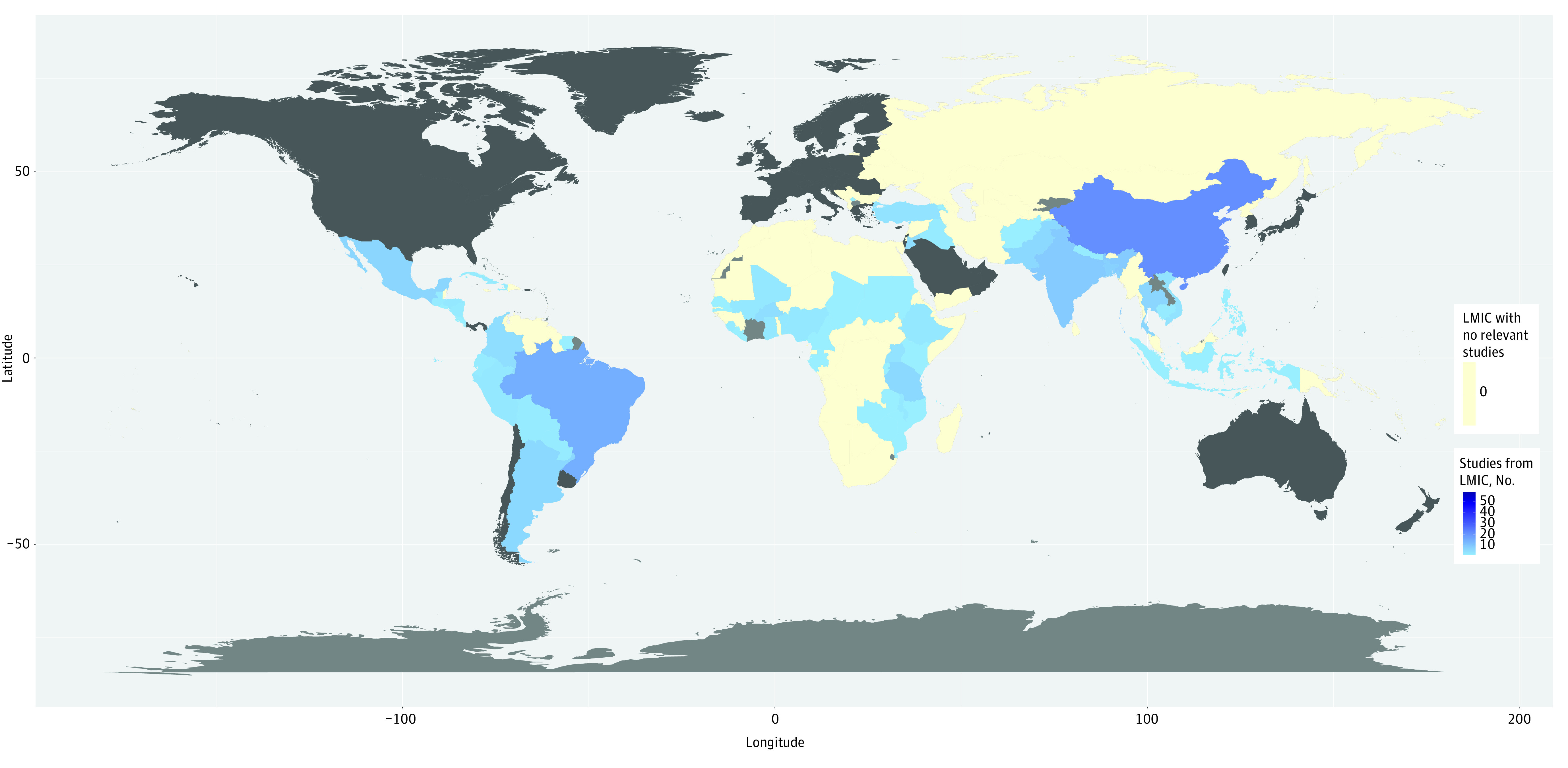
Studies of Overuse of Surgical Procedures in Low- and Middle-Income Countries (LMICs)

The 133 included studies collectively reported on more than 9.1 million surgical procedures (median per study, 894 [IQR, 97-4259]) and more than 11.4 million participants (median per study, 989 [IQR, 257-6857]) from 63 LMICs.^9,11,16,17,20,38,39,40,41,42,43,44,45,46,47,48,49,50,51,52,53,54,55,56,57,58,59,60,61,62,63,64,65,66,67,68,69,70,71,72,73,74,75,76,77,78,79,80,81,82,83,84,85,86,87,88,89,90,91,92,93,94,95,96,97,98,99,100,101,102,103,104,105,106,107,108,109,110,111,112,113,114,115,116,117,118,119,120,121,122,123,124,125,126,127,128,129,130,131,132,133,134,135,136,137,138,139,140,141,142,143,144,145,146,147,148,149,150,151,152,153,154,155,156,157,158,159,160,161,162,163,164,165^ Of all 133 LMICs, we found studies from 15 of all 29 low-income countries (51.7%),^11,16,17,38,39,64,68,69,70,71,92,100,103,105,129,143^ 25 of all 50 lower-middle–income countries (50.0%),^41,44,45,48,53,55,56,57,58,63,67,72,77,80,81,83,84,96,98,104,107,108,112,115,116,120,122,123,124,126,127,130,137,140,153,155,164,166^ and 23 of all 55 upper-middle–income countries (41.8%).^9,40,43,49,50,51,54,59,61,62,66,73,74,75,76,79,82,85,86,87,88,89,90,91,93,95,97,99,101,102,106,109,110,111,113,114,117,118,121,125,128,131,132,134,135,136,138,139,141,142,144,149,151,152,154,156,157,158,159,161,162,165^ Fourteen studies (10.5%) were multinational.^11,16,20,39,46,47,64,65,70,78,133,143,160,162^ Of the 119 studies (89.5%) originating from single countries, 69 (58.0%) were from upper-middle–income countries^9,11,20,40,42,43,47,49,50,51,52,54,59,60,61,62,66,73,74,75,76,79,82,85,86,87,88,89,90,91,93,95,97,99,101,102,106,109,110,111,113,114,117,118,121,125,128,131,132,134,135,136,138,139,141,142,144,145,146,147,148,149,151,152,154,156,157,158,159,160,161,163,164,165^ and 30 (25.2%) were from East Asia and the Pacific^40,42,49,50,52,74,75,76,82,87,94,95,102,110,113,114,117,118,135,138,144,148,149,150,151,156,157,158,159,163^ (Table 1 and Figure 2).

**Table 1.** Characteristics of Included Studies

Characteristic	Studies, No. (%) (N = 133)
Publication year	
Before 2010	18 (13.5)
2010-2020	93 (70.0)
2021 or later	22 (16.5)
Language of publication	
English	126 (94.7)
Spanish	3 (2.3)
Farsi	1 (0.8)
Dutch	1 (0.8)
Chinese	1 (0.8)
Portuguese	1 (0.8)
Single country	
All	119 (89.5)
Country income level, No./total No. (%)	
Low	11/119 (9.2)
Lower middle	39/119 (32.8)
Upper middle	69/119 (58.0)
WHO region, No./total No. (%)	
Sub-Saharan Africa	19/119 (16.0)
East Asia and Pacific	30/119 (25.2)
Europe and Central Asia	6/119 (5.0)
Latin America and the Caribbean	20/119 (16.8)
Middle East and North Africa	21/119 (17.6)
South Asia	19/119 (16.0)
Multiple countries	14 (10.5)
Study design	
Interventional	9 (6.8)
Randomized clinical trial	4 (3.0)
Controlled or before-and-after study	5 (3.8)
Observational	107 (80.5)
Cross-sectional	68 (51.1)
Cohort, prospective or retrospective	39 (29.3)
Other	11 (8.3)
Secondary research	6 (4.5)
Health care setting	
Hospital-based or secondary care	93 (69.9)
Home-based or community or primary care	12 (9.0)
Mixed	11 (8.3)
Unclear or not applicable	17 (12.8)
Analysis approach	
Quantitative	93 (69.9)
Qualitative	17 (12.8)
Mixed	23 (17.3)
Condition or system treated^a^	
Maternal, including genitourinary	115 (86.5)
Trauma	4 (3.0)
Cardiovascular	5 (3.8)
Cancer	2 (1.5)
Gastrointestinal tract	4 (3.0)
Respiratory tract	1 (0.8)
Surgical indication	
Emergency	13 (9.8)
Elective	19 (14.3)
Unclear	34 (25.6)
Mixed	67 (50.4)
Severity of surgical procedure	
Major	128 (96.6)
Minor	5 (3.4)
Type of surgical procedure	
Open	125 (94.0)
Keyhole	3 (2.3)
Unclear	5 (3.8)
Surgical specialty	
Gynecology and obstetrics	115 (86.5)
General surgery	7 (5.3)
Surgical oncology	4 (3.0)
Cardiovascular surgery	4 (3.0)
Unclear	3 (2.3)

Abbreviation: WHO, World Health Organization.

^a^ Categories are not mutually exclusive.

Most studies (115 [86.5%]) were published in 2010 or after,^9,11,16,17,20,38,39,40,41,43,44,45,46,47,54,56,57,58,59,60,61,62,63,66,67,68,69,71,72,75,77,78,79,80,81,82,83,84,85,87,88,89,90,92,93,94,95,96,97,98,99,100,101,102,103,104,105,106,107,108,109,111,112,113,114,115,116,117,118,119,120,121,123,124,125,126,127,128,129,131,132,133,134,135,136,137,138,139,140,141,142,143,144,145,146,147,149,150,151,154,156,157,158,159,160,161,162,163,164^ with a marked increase in the number of included studies per year. Most articles were written in English (126 [94.7%]).^9,11,16,17,20,38,39,40,41,42,43,44,45,46,47,48,49,50,51,52,53,54,55,56,57,58,59,60,62,63,64,65,66,67,68,69,70,71,72,74,75,77,78,79,80,81,82,83,84,85,86,87,88,89,90,91,92,93,94,95,96,98,99,100,101,102,103,104,105,106,107,108,109,110,111,112,113,114,115,116,117,118,119,120,121,122,123,124,125,126,127,129,130,131,132,134,135,136,137,138,140,141,142,143,144,145,146,147,148,149,150,151,152,153,154,155,156,157,158,159,160,161,162,163,164,166^ Health care settings were hospital based or secondary care in 93 studies (69.9%).^9,17,38,40,41,42,43,44,45,49,50,51,55,58,61,62,64,65,67,68,69,70,73,74,75,76,77,79,81,82,84,86,90,92,93,94,95,98,99,100,101,102,103,104,105,106,107,108,109,110,111,113,114,115,116,118,119,120,122,123,124,125,126,127,128,129,130,131,132,134,135,136,138,139,141,142,143,147,148,149,150,151,152,153,155,156,158,159,160,161,162,163,164^ Of the 133 included studies, 107 (80.5%) were observational^9,11,38,39,40,41,42,43,44,45,47,48,49,50,51,53,54,55,56,57,58,59,60,61,62,63,64,66,67,69,70,71,72,73,74,75,76,79,80,81,82,83,84,85,86,87,88,89,90,92,93,94,95,96,97,98,100,101,102,104,105,106,108,109,110,111,112,113,114,117,118,119,120,121,122,123,124,125,126,128,129,130,131,133,134,135,136,137,138,139,141,142,143,144,147,148,151,152,153,154,155,156,157,158,159,162,164^ and 17 (12.8%) were qualitative.^46,62,70,77,78,87,90,99,100,116,124,127,143,149,150,156,160^ Most of the included studies (115 [86.5%]) had a gynecology and obstetrics focus^9,11,16,17,20,38,39,45,46,47,48,49,50,51,52,53,54,55,56,57,58,59,60,62,63,64,65,66,67,68,69,70,71,72,73,74,76,77,78,79,80,81,82,83,84,85,86,87,88,89,90,91,92,93,94,95,96,97,98,99,101,102,103,104,105,106,109,110,111,112,113,114,115,116,117,118,120,121,124,125,126,127,128,129,130,131,132,134,135,136,137,139,140,141,142,143,144,145,146,147,148,149,150,151,155,156,157,158,159,160,161,162,163,164,166^ (Table 1 and eTable in Supplement 1).

### Estimates of Extent of Overuse of Surgical Procedures in LMICs

Of the 133 included studies, 42 (31.6%) reported on the extent of overuse of surgical procedures in LMICs.^9,11,38,39,40,41,42,44,49,56,58,60,61,62,64,70,73,74,76,81,84,85,87,89,94,98,104,105,115,119,120,125,126,132,133,139,141,143,153,155,158,166^ Most of these reported on the extent of unnecessary CDs (36 [85.7%]).^9,11,38,39,49,56,58,60,62,64,70,73,74,76,81,84,85,87,89,94,98,104,105,115,119,120,125,126,132,133,139,141,143,155,158,166^

#### CD

Estimated rates of unnecessary CDs in LMICs reported in included studies ranged from 12% in a retrospective study of 300 women with low-risk pregnancies who underwent CD at 10 referral hospitals in Burkina Faso^38^ to 81% in a cross-sectional study of 416 primary CDs in 4 hospitals in Colombia.^165^ Estimated rates of unnecessary CDs were between 19.5% and 50% in 18 (50.0%) of the 36 included studies on CDs^11,60,62,73,74,85,87,98,104,105,115,120,132,133,141,143,155,158^ (Table 2, Table 3). A secondary analysis of data from 63 LMICs showed substantial within-country economic inequalities, with CD rates tending to be lower in lower-income areas, likely representing underuse, and higher in higher-income areas, often representing overuse.^39^

**Table 2.** Examples of Key Findings Grouped by the Main Themes Addressed^a^

Study	Description	Key findings
**Estimation of overuse of surgical procedures (n = 42)**		
Belizán et al,^70^ 1999	Ecological study across 19 Latin American countries to estimate the incidence of CD and factors associated with it	Of the 19 countries, 12 had CD rates >15% (range, 16.8%-40%), with >850 000 unnecessary CDs performed each year in Latin America. Higher CD rates were observed in private than public hospitals and in countries with higher gross national product per capita.
Boatin et al,^39^ 2018	Observational study of 72 LMICs to estimate inequalities in CD rates between and within countries	Rates of CD ranged from 0.6% in South Sudan to 58.9% in the Dominican Republic. Substantial within-country economic inequalities in CDs remain (rates were lowest in the one-fifth lowest income [median, 3.7%] and highest in the one-fifth highest income [median, 18.4%]). These inequalities might be due to inadequate access to emergency obstetric care among the lowest-income subgroups and high numbers of CDs without medical indication in the highest-income subgroups, especially in middle-income countries.
Lin et al,^40^ 2020	Prospective multicenter cohort study to evaluate the appropriateness of coronary revascularization among 6085 patients with coronary heart disease	Only 1617 of the patients (26.6%) were deemed appropriate for coronary revascularization.
**Factors associated with overuse of surgical procedures (n = 60)**		
Singh et al,^53^ 2018	Analysis of 24 398 deliveries in 19 states in India to examine factors associated with CD	Rates of CD were higher in the private vs public health care sector (37.9% vs 13.7%; OR, 3.8 [95% CI, 3.1-4.7]).
Hatemleh et al,^99^ 2019	Qualitative study of 35 first-time mothers requesting elective CD in a private hospital in Jordan	The lived negative experience of the social network was a major influence on the women’s CD decision. Main themes were fear of vaginal birth process, concerns about future sexual experiences, and the need for humanized birth.
Colomar et al,^78^ 2021	Systematic review of 52 qualitative studies from 28 countries on women’s views and perspectives about CD	Major factors contributing to the women’s preferences for CD in the absence of medical indications included fear of pain, uncertainty about vaginal birth, and positive views about CD.
**Consequences of overuse of surgical procedures (n = 8)**		
Gonzalez-Perez et al,^54^ 2001	Analysis of the economic costs of excess CDs using national data on 2 532 762 deliveries over 5 y in Mexico	A conservative estimate of the economic cost of unnecessary CDs in public health care institutions in Mexico was US $12 204 774.
Hu et al,^49^ 2016	Analysis to estimate the excess economic burden of unnecessary CDs among 33 476 deliveries from 17 randomly selected hospitals in Beijing, China	Costs of unnecessary CDs were, on average, US $472 higher than that of vaginal deliveries. The total excess economic burden caused by unnecessary CDs was estimated as US $38.97 million for Beijing and US $3.29 billion across China in 2011, equivalent to the annual health expenditure of >139 575 residents in Beijing and >11 783 120 residents in China.
Haider et al,^96^ 2018	Economic analysis of the economic burden of unnecessary CDs in Bangladesh	Rates of CDs increased from 33% in 2000 to 63% in 2014. Delivery costs accounted for 10.3% of the total health expenditure; CD costs accounted for 6.9% of the total health expenditure.
**Solutions for the problem of overuse of surgical procedures (n = 23)**		
Kaboré et al,^17^ 2019	Cluster RCT evaluating effects of a multifaceted intervention (on-site training; audit, feedback, and reminders; and clinical algorithms) on the rate of unnecessary CDs in 22 referral hospitals in Burkina Faso	The intervention resulted in a clinically important reduction in the rate of unnecessary CDs (adjusted OR, 0.22 [95% CI, 0.14-0.34]; adjusted risk difference, −17.0% [95% CI, −19.2 to −13.2]), with no significant differences in maternal or neonatal deaths between groups.
Torloni et al,^16^ 2020	Systematic review of 7 mass media campaigns between 2009 and 2017, mostly in LMICs, to reduce the rate of unnecessary CDs	There was a paucity of mass media campaigns to reduce unnecessary CDs. Most assessed outcomes for knowledge, but none assessed outcomes for CD rates.
Karthikeyan et al,^107^ 2017	A before-and-after study evaluating the implementation of appropriateness-based reimbursement of elective invasive coronary procedures in 106 hospitals in 8 districts covering a population of 20 424 585 individuals in India	There was a reduction of 12.3% (95% CI, 8.9%-15.5%) in procedures performed, with similar reductions in public and private hospitals. At current rates, use of appropriateness-based reimbursement would result in potential annual savings of about ₹57 million (US $930 000; 95% CI, US $570 000 to US $1 300 000).

Abbreviations: CD, cesarean delivery; LMICs, low- and middle-income countries; OR, odds ratio; RCT, randomized clinical trial.

^a^ Results reported in the table are based on key studies selected to represent different countries for the main theme or focus (eg, solution or estimate). However, these studies were conducted in local settings and might not be representative of the wider LMIC context. Therefore, generalizability of these findings to the LMIC context is limited, and generalizations should be made with caution.

**Table 3.** Summary of the Key Findings of Included Studies

Study topic	Key findings	Country income level	WHO region
**Estimated rate of overuse of surgical procedures (n = 42)**			
CD (n = 36)	12%-81%, 50% of estimates between 19.5% and 50%	2 Low, 12 lower middle, 16 upper middle, and 6 multiple countries	6 SSA, 5 MENA, 5 SA, 5 EAP, 8 LAC, 1 Europe, and 6 multiple countries
Percutaneous coronary revascularization (n = 3)	4%-60%	1 Lower middle, 2 upper middle	1 EAP, 1 LAC, and 1 SA
Operative management for blunt abdominal trauma (n = 1)	25%	1 Lower middle	1 SSA
Appendectomy (n = 1)	18%	1 Lower middle	1 SA
Chest tube insertion (n = 1)	13%	1 Upper middle	1 EAP
**Factors associated with overuse of surgical procedures (n = 60)**			
Individual level (n = 14)			
Clinicians (n = 5)	Examples: limited training and skills and inadequate awareness of guidelines	2 Lower middle, 3 upper middle	1 MENA, 1 SA, 1 EAP, and 2 LAC
Patients and the public (n = 9)	Examples: maternal request for CD and fear of or concerns about vaginal birth	4 Lower middle, 4 upper middle, and 1 multiple countries	1 SSA, 4 MENA, 1 SA, 2 EAP, and 1 multiple countries
System level (n = 18)			
Policy and regulations (n = 2)	Example: birth control policy	2 Upper middle	2 EAP
Resources (n = 8)	Example: lack or limited availability of resources such as pain control and staff	2 Low, 2 lower middle, and 4 upper middle	2 SSA, 2 MENA, 1 SA, 2 EAP, and 1 LAC
Financial (n = 8)	Example: private vs public health insurance or facilities	1 Low, 3 lower middle, and 4 upper middle	2 SSA, 2 SA, 2 EAP, 1 LAC, and 1 Europe
Both (n = 28)			
Resources (system level) and clinicians and patients (individual level) (n = 10)	Examples: inadequate awareness of guidelines, maternal request for CD, limited availability of resources	4 Lower middle, 3 upper middle, and 3 multiple countries	1 SSA, 2 MENA, 2 SA, 1 EAP, 1 LAC, and 3 multiple countries
Resources (system level) and clinicians (individual level) (n = 5)	Examples: limited training and skills and lack or limited availability of resources	1 Low, 1 lower middle, and 3 upper middle	2 SSA, 1 EAP, 1 LAC, and 1 Europe
Financial (system level) and patients (individual level) (n = 13)	Examples: maternal request for CD and private vs public health insurance or facilities	9 Lower middle, 4 upper middle	7 SA, 3 EAP, 1 MENA, and 2 LAC
**Consequences of overuse of surgical procedures (n = 8)**			
Economic consequences (n = 5)	NA	1 Lower middle, 4 upper middle	1 SA, 2 EAP, 1 MENA, and 1 LAC
Complications and adverse events (n = 3)	NA	3 Upper middle	2 EAP, 1 MENA
**Solutions for the problem of overuse of surgical procedures (n = 23)**			
Community level (n = 5)	Example: mass media campaign	4 Upper middle, 1 multiple countries	3 MENA, 1 LAC, and 1 multiple countries
Policy level (n = 6)	Example: introduction of regulations and guidelines	1 Low, 1 lower middle, 3 upper middle, and 1 multiple countries	2 SA, 1 SSA, 1 MENA, 1 Europe, and 1 multiple countries
Individual level, patients and the public (n = 3)	Example: text messaging interventions	3 Upper middle	1 EAP, 2 LAC
Individual level, clinicians (n = 4)	Example: provisions of training interventions	1 Lower middle, 2 upper middle, and 1 multiple countries	2 MENA, 1 SA, and 1 multiple countries
Multifaceted (n = 5)	Example: audit feedback and training and provision of guidelines	2 Low, 2 upper middle, and 1 multiple countries	2 SSA, 2 EAP, and 1 multiple countries

Abbreviations: CD, cesarean delivery; EAP, East Asia Pacific; LAC, Latin America and Caribbean; MENA, Middle East and North Africa; NA, not applicable; SA, South Asia; SSA, Sub-Saharan Africa; WHO, World Health Organization.

#### Non-CD Literature

Of the 6 studies reporting non-CD surgical procedures,^40,41,42,44,61,153^ 3 (50.0%) reported estimates of unnecessary percutaneous coronary revascularization.^40,41,61^ For instance, Patil et al^41^ examined the appropriateness of 894 percutaneous coronary revascularizations (with insertion of stents) in a large tertiary hospital in India and found that only 39.4% were deemed appropriate. The remaining 3 studies (50.0%) reported on various topics related to trauma surgery^42^ and abdominal surgery.^44,153^ For instance, an analysis of 408 patients diagnosed with acute appendicitis found that 72 (17.6%) had unnecessary appendectomy.^44^

### Associated Factors

Of the 133 included studies, 60 (45.1%) reported on factors associated with overuse of surgical procedures (primarily CD) in LMICs.^43,45,46,47,48,53,57,59,63,66,67,69,71,72,77,78,79,80,82,83,86,92,93,95,99,100,101,102,106,109,112,113,114,116,117,118,122,123,124,127,128,129,130,134,135,136,137,140,144,145,146,147,148,149,150,156,157,159,162,164^ A total of 14 studies (23.3%) reported on individual-level factors (ie, clinician or patient)^45,46,48,59,66,72,82,93,99,124,134,157,159,162^; 18 (30.0%), system-level factors (ie, institutional or organizational)^71,83,86,92,100,101,113,114,116,117,127,128,130,135,145,148,156,164^; and 28 (46.7%), both individual- and system-level factors^43,47,53,57,63,67,69,77,78,79,80,95,102,106,109,112,118,122,123,129,136,137,140,144,146,147,149,150^ (Table 3).

#### Individual-Level Factors

A study of 4357 deliveries and interviews with 275 clinicians in 13 public hospitals in 4 governorates in Egypt found that a convenience incentive, lack of supervision and training, and absence of familiarity with clinical guidelines were important factors associated with unnecessary CDs.^45^ In-depth interviews with 25 clinicians, patients, and policy makers in Benin and Mali found that inappropriate use of CD was particularly alarming in countries with high fertility, as it poses a threat to the mothers and infants in the short term (current pregnancy and delivery) and long term (subsequent pregnancies).^46^ The main factors reported were maternal fear and pain, lack of resources, staff suffering, and ethical breakdowns.^46^

#### System-Level Factors

An ecological study across 172 countries found that private health financing was positively associated with proportions of unnecessary CDs, with each 10% increase in out-of-pocket expenditure associated with a 0.7% increase in proportions of unnecessary CDs.^47^ An analysis of factors associated with unnecessary CDs among 4903 women in Bangladesh also found higher odds of unnecessary CDs in private vs public health facilities (odds ratio [OR], 10.35; 95% CI, 8.55-12.54)^48^ (Table 2).

### Consequences

Consequences of overuse of surgical procedures were reported in only 8 studies (6.0%).^49,50,54,96,138,142,151,154^ The most frequently reported consequence was the economic burden. For example, an analysis of 33 476 deliveries in China estimated the annual cost of unnecessary CDs to be US $38.97 million for Beijing and US $3.29 billion across China in 2011.^49^ Complications following surgical procedures have also been reported. For example, a secondary analysis of a hospital-based database of pregnant women and newborns in Thailand found a positive correlation between the increasing rates of unnecessary CDs and rates of adverse maternal and neonatal outcomes.^50^ For non-CD literature, a 5-year longitudinal analysis of the consequences of 1073 unnecessary appendectomies in Iraq reported a complication rate of around 3% (eg, wound infection and septicemia) and 0.5% mortality.^43^

### Potential Solutions

A total of 23 studies (17.3%) reported on evaluations of potential solutions,^16,17,20,51,52,65,68,75,88,90,91,97,103,107,108,110,111,121,131,152,160,161,163^ and 9 of those studies (39.1%) were interventional rather than observational^17,52,65,68,91,103,107,161,163^ (eTable in Supplement 1). A controlled study of 350 first-time pregnant women planning a cesarean birth without any medical indications found that a social media campaign called “B Butterfly” that promoted vaginal delivery was associated with a substantial reduction in unnecessary CDs (64.4% vs 35.6%).^51^ Similarly, a controlled trial of 2115 pregnant women in rural China found that sending pregnant women short informational messages with advice regarding both care seeking and good home prenatal practices was associated with a reduction in unnecessary CDs (OR, 0.66; 95% CI, 0.49-0.90).^52^ Another example is a cluster randomized clinical trial of 22 referral hospitals in Burkina Faso, a low-income country, which showed that a multifaceted intervention (on-site training, audit and feedback, reminders, and clinical algorithms) resulted in a clinically important reduction in the rate of unnecessary CDs (−17.0%; 95% CI, −19.2% to −13.2%).^17,167^

## Discussion

Our systematic scoping review found evidence of overuse of surgical procedures in many LMICs; unnecessary CDs were the most prevalent example, with estimated rates ranging from 12% to 81%. Factors associated with overuse included lack of training and supervision, limited resources and staffing, financial incentives and profit motive, and social and professional norms. Major consequences reported were high costs of unnecessary surgeries, most of which were CDs, and surgical complications. Our review identified a few practical, effective solutions to address the problem of unnecessary surgical procedures, chiefly delivering evidence-based information.

The majority of the studies included in our review were published within the past decade, indicating a growing interest in understanding and addressing the problem of overuse of surgical procedures in LMICs. We observed substantial variations in the rates of overuse of surgical procedures that can be attributed to population-related (eg, country and setting) and study-related (eg, sampling frame) differences. Few studies explored associated factors and consequences or evaluated potential solutions for the problem of overuse of surgical procedures. A key factor associated with overuse was private health financing, identified in 2 large studies.^53,71^ Key consequences were cost and waste, featured in an analysis of over 2.5 million deliveries over 5 years in Mexico.^54^ These consequences have severe implications for health care systems in LMICs, which are already fragmented and vulnerable.^166^ We found a few studies^167,168,169^ that evaluated innovative and ultimately effective solutions to reduce unnecessary CDs in LMICs. For example, multifaceted interventions including on-site training, audit and feedback, reminders, and clinical algorithms resulted in a clinically important 17.0% reduction in the rate of unnecessary CDs.^167^ Frameworks have been established to evaluate and implement these multifaceted interventions and to ensure achieving the intended outcomes while addressing contextual barriers in LMICs.^168,169^

In general, the results of our scoping review suggest a limited amount of literature addressing the issue of surgical procedure overuse compared with the abundance of literature some of us discovered in previous scoping reviews on overdiagnosis and overuse of tests and medications.^31,32^ This suggests either that the problem of overuse of surgical procedures may be not as substantial in places where limited access and underuse of health care services is clearly a priority (which might pose a marked inequity issue) or that the problem of overuse of surgery is understudied or underreported. Evidence from high-income countries showed that several surgical procedures were unnecessary.^37,170^ For example, an umbrella review of common elective orthopedic procedures found no high-quality evidence to support the effectiveness of commonly recommended elective orthopedic procedures compared with nonoperative alternatives.^37^ An analysis of surgical procedures in the UK National Health Services identified 6 unnecessary, low-value surgical procedures: spinal surgery for lower back pain, myringotomy to relieve eardrum pressure, inguinal hernia repair, cataract removal, primary hip replacement, and hysterectomy for heavy menstrual bleeding.^170^

The findings suggest the need for research in this field to expand its focus beyond CDs to examine the extent of and factors associated with overuse of other surgical procedures and explore potential consequences. The results also suggest the need for evaluation and implementation of potential solutions to reduce unnecessary surgical procedures, as exemplified by the ongoing WHO trial to reduce unnecessary CDs,^12^ while simultaneously enhancing access to essential surgical procedures when required, with a focus on ensuring equitable distribution of limited resources.

### Limitations

An important limitation of our review is the inherent variability in the definitions and methods used to define overuse of surgical procedures across the included studies. We accepted the definitions of *overuse* and *low value* used by the authors of included studies. The broad scope of our review required us to encompass studies using diverse definitions and measurement approaches. Other limitations are the subjective categorization of associated factors into system and individual levels as well as the use of the 2021 World Bank income level list for studies conducted in previous years, although income levels have remained mostly unchanged for most LMICs. Despite these limitations, our scoping review provides insights into the existing literature on overuse of surgical procedures and contributes to the identification of gaps and areas for future research. Careful consideration of these limitations is crucial when interpreting the findings and applying them to decision-making processes.

## Conclusions

This study found growing evidence of overuse of surgical procedures in LMICs, which generates significant harm and waste, with unnecessary CDs being the most commonly studied problem. A better understanding of the problems and robust evaluation of solutions are needed. Addressing underuse of and limited access to surgical procedures in LMICs may benefit from pragmatic efforts to reduce the waste of resources from the overuse of unnecessary surgical procedures. Reducing unnecessary, low-value interventions and prioritizing high-value surgical procedures should help address these interconnected issues and ensure equitable, sustained access to surgical procedures in LMICs, which has gained importance as worldwide priorities.

## Notes

Supplement 1. **eAppendix 1.** 2021 World Bank Categorizations of Low-Income, Lower-Middle Income, Upper-Middle Income, and High-Income Countries **eAppendix 2.** Search Strategy Used to Identify Studies **eTable.** Characteristics of Included Studies in the Scoping Review
eReferences

Supplement 2.
Data Sharing Statement
