# Supplementary material for: Low-Value Surgical Procedures in Low- and Middle-Income Countries: A Systematic Scoping Review
Source: JAMA Netw Open. 2023 Nov 7;6(11):e2342215. doi: 10.1001/jamanetworkopen.2023.42215 (PMC10630901; doi:10.1001/jamanetworkopen.2023.42215)
Supplement: Supplement 1. — eAppendix 1. 2021 World Bank Categorizations of Low-Income, Lower-Middle Income, Upper-Middle Income, and High-Income Countries eAppendix 2. Search Strategy Used to Identify Studies eTable. Characteristics of Included Studies in the Scoping Review eReferences [file jamanetwopen-e2342215-s001.pdf]

Supplementary Online Content

Albarqouni L, Abukmail E, MohammedAli M, et al. Low-value surgical procedures in low- and middle-income countries: a systematic scoping review. *JAMA Netw Open*. 2023;6(11):e2342215. doi:10.1001/jamanetworkopen.2023.42215

eAppendix 1. 2021 World Bank Categorizations of Low-Income, Lower-Middle Income, Upper-Middle Income, and High-Income Countries

eAppendix 2. Search Strategy Used to Identify Studies

eTable. Characteristics of Included Studies in the Scoping Review

eReferences

This supplementary material has been provided by the authors to give readers additional information about their work.

© 2023 Albarqouni L et al. *JAMA Network Open*.

eAppendix 1. The 2021 World Bank categorisations of countries per economy: low income, lower-middle income, upper-middle income, and high-income countries.

LOW-INCOME ECONOMIES ($1,045 OR LESS) [27] (INCLUDED)

Afghanistan
Burkina Faso
Burundi
Central African Republic
Chad
Congo, Dem. Rep
Eritrea
Ethiopia
Gambia, The
Guinea
Guinea-Bissau
Korea, Dem. People's Rep.
Liberia
Madagascar
Malawi
Mali
Mozambique
Niger
Rwanda
Sierra Leone
Somalia
South Sudan
Sudan
Syrian Arab Republic
Togo
Uganda
Yemen, Rep.

LOWER-MIDDLE INCOME ECONOMIES ($1,046 TO $4,095)[55] (INCLUDED)

Angola
Algeria
Bangladesh
Belize
Benin
Bhutan
Bolivia
Cabo Verde
Cambodia
Cameroon
Comoros
Congo, Rep.
Côte d'Ivoire
Djibouti
Egypt, Arab Rep.
El Salvador
Eswatini
Ghana
Haiti
Honduras
India
Indonesia
Iran, Islamic Rep
Kenya
Kiribati
Kyrgyz Republic
Lao PDR
Lesotho
Mauritania
Micronesia, Fed. Sts.
Mongolia
Morocco
Myanmar
Nepal
Nicaragua
Nigeria
Pakistan
Papua New Guinea
Philippines
Samoa
São Tomé and Principe
Senegal
Solomon Islands
Sri Lanka
Tanzania
Tajikistan
Timor-Leste
Tunisia
Ukraine
Uzbekistan
Vanuatu
Vietnam
West Bank and Gaza
Zambia
Zimbabwe

UPPER-MIDDLE-INCOME ECONOMIES ($4,096 TO $12,695) [55] (INCLUDED)

Albania
American Samoa
Argentina
Armenia
Azerbaijan
Belarus
Bosnia and Herzegovina
Botswana
Brazil
Bulgaria
China
Colombia
Gabon
Georgia
Grenada
Guatemala
Guyana
Iraq
Jamaica
Jordan
Kazakhstan
Kosovo
Lebanon
Libya
Namibia
North Macedonia
Panama
Paraguay
Peru
Romania
Russian Federation
Serbia
South Africa
St. Lucia
St. Vincent and the Grenadines
Suriname

© 2023 Albarqouni L et al. *JAMA Network Open*.

Costa Rica	Malaysia	Thailand
Cuba	Maldives	Tonga
Dominica	Marshall Islands	Turkey
Dominican Republic	Mauritius	Turkmenistan
Equatorial Guinea	Mexico	Tuvalu
Ecuador	Moldova	
Fiji	Montenegro	

HIGH-INCOME ECONOMIES ($12,696 OR MORE)[80](EXCLUDED)

Andorra	Greece	Poland
Antigua and Barbuda	Greenland	Portugal
Aruba	Guam	Puerto Rico
Australia	Hong Kong SAR, China	Qatar
Austria	Hungary	San Marino
Bahamas, The	Iceland	Saudi Arabia
Bahrain	Ireland	Seychelles
Barbados	Isle of Man	Singapore
Belgium	Israel	Sint Maarten (Dutch part)
Bermuda	Italy	Slovak Republic
British Virgin Islands	Japan	Slovenia
Brunei Darussalam	Korea, Rep.	Spain
Canada	Kuwait	St. Kitts and Nevis
Cayman Islands	Latvia	St. Martin (French part)
Channel Islands	Liechtenstein	Sweden
Chile	Lithuania	Switzerland
Croatia	Luxembourg	Taiwan, China
Curaçao	Macao SAR, China	Trinidad and Tobago
Cyprus	Malta	Turks and Caicos Islands
Czech Republic	Monaco	United Arab Emirates
Denmark	Nauru	United Kingdom
Estonia	Netherlands	United States
Faroe Islands	New Caledonia	Uruguay
Finland	New Zealand	Virgin Islands (U.S.)
France	Northern Mariana Islands	
French Polynesia	Norway	
Germany	Oman	
Gibraltar	Palau	

© 2023 Albarqouni L et al. *JAMA Network Open*.

eAppendix 2. Search strategy used to identify studies on PubMed; Embase; PsycINFO; Global Index Medicus on 21/10/2021

PubMed

("Medical Overuse"[Mesh] OR Overmedicalization[tiab] OR Overmedicalisation[tiab] OR Overtreatment[tiab] OR "Over-treatment"[tiab] OR Overuse[tiab] OR Unnecessary[tiab] OR Unwarranted[tiab] OR Inappropriate[tiab] OR De-implementation[tiab] OR Deimplementation[tiab] OR Low-value care [tiab] OR low-value hospital care [tiab] OR low-value healthcare [tiab] OR wasteful care [tiab] OR wasteful healthcare [tiab] OR wasteful hospital care [tiab] OR overuse of healthcare [tiab] OR overuse procedure* [tiab] OR medical overuse [tiab] OR inappropriate healthcare [tiab] OR inappropriate care [tiab] OR unwanted healthcare [tiab] OR unwanted care [tiab] OR unnecessary healthcare [tiab] OR unnecessary care [tiab])

AND

("Surgical Procedures, Operative"[Mesh] OR "surgery"[sh] OR "Cesarean Section"[Mesh] OR "Arthroplasty"[Mesh] OR "Arthroscopy"[Mesh] OR "Spinal Fusion"[Mesh] OR Surgery[tiab] OR Surgeries[tiab] OR Surgical[tiab] OR Caesarean[tiab] OR operation[tiab] OR operative[tiab] OR operations[tiab] OR Arthroscopic[tiab] OR Arthroscopies[tiab] OR Arthroscopy[tiab] OR carpal tunnel surgery[tiab] OR carpal tunnel release[tiab] OR spinal decompression[tiab])

AND

(afghanistan[Text Word] OR albania[Text Word] OR algeria[Text Word] OR american samoa[Text Word] OR angola[Text Word] OR antigua[Text Word] OR barbuda[Text Word] OR argentina[Text Word] OR armenia[Text Word] OR armenian[Text Word] OR aruba[Text Word] OR azerbaijan[Text Word] OR bahrain[Text Word] OR bangladesh[Text Word] OR barbados[Text Word] OR belarus[Text Word] OR byelarus[Text Word] OR belorussia[Text Word] OR byelorussian[Text Word] OR belize[Text Word] OR british honduras[Text Word] OR benin[Text Word] OR dahomey[Text Word] OR bhutan[Text Word] OR bolivia[Text Word] OR bosnia[Text Word] OR herzegovina[Text Word] OR botswana[Text Word] OR bechuanaland[Text Word] OR brazil[Text Word] OR brasil[Text Word] OR bulgaria[Text Word] OR burkina faso[Text Word] OR burkina fasso[Text Word] OR upper volta[Text Word] OR burundi[Text Word] OR urundi[Text Word] OR cabo verde[Text Word] OR cape verde[Text Word] OR cambodia[Text Word] OR kampuchea[Text Word] OR khmer republic[Text Word] OR cameroon[Text Word] OR cameron[Text Word] OR cameroun[Text Word] OR central african republic[Text Word] OR ubangi shari[Text Word] OR chad[Text Word] OR chile[Text Word] OR china[Text Word] OR colombia[Text Word] OR comoros[Text Word] OR comoro islands[Text Word] OR mayotte[Text Word] OR congo[Text Word] OR zaire[Text Word] OR costa rica[Text Word] OR cote d'ivoire[Text Word] OR cote d' ivoire[Text Word] OR cote divoire[Text Word] OR cote d ivoire[Text Word] OR ivory coast[Text Word] OR croatia[Text Word] OR cuba[Text Word] OR cyprus[Text Word] OR czech republic[Text Word] OR czechoslovakia[Text Word] OR djibouti[Text Word] OR french somaliland[Text Word] OR dominica[Text Word] OR dominican republic[Text Word] OR ecuador[Text Word] OR egypt[Text Word] OR united arab republic[Text Word] OR el salvador[Text Word] OR equatorial guinea[Text Word] OR spanish guinea[Text Word] OR eritrea[Text Word] OR estonia[Text Word] OR eswatini[Text Word] OR swaziland[Text Word] OR ethiopia[Text Word] OR fiji[Text Word] OR gabon[Text Word] OR gabonese republic[Text Word] OR gambia[Text Word] OR georgia[Text Word] OR georgian[Text Word] OR ghana[Text Word] OR gold coast[Text Word] OR gibraltar[Text Word] OR greece[Text Word] OR grenada[Text Word] OR guam[Text Word] OR guatemala[Text Word] OR guinea[Text Word] OR guyana[Text Word] OR guiana[Text Word] OR haiti[Text Word] OR hispaniola[Text Word] OR

honduras[Text Word] OR hungary[Text Word] OR india[Text Word] OR indonesia[Text Word] OR timor[Text Word] OR iran[Text Word] OR iraq[Text Word] OR isle of man[Text Word] OR jamaica[Text Word] OR jordan[Text Word] OR kazakhstan[Text Word] OR kazakh[Text Word] OR kenya[Text Word] OR korea[Text Word] OR kosovo[Text Word] OR kyrgyzstan[Text Word] OR kirghizia[Text Word] OR kirgizstan[Text Word] OR kyrgyz republic[Text Word] OR kirghiz[Text Word] OR laos[Text Word] OR lao pdr[Text Word] OR lao people's democratic republic[Text Word] OR latvia[Text Word] OR lebanon[Text Word] OR lesotho[Text Word] OR basutoland[Text Word] OR liberia[Text Word] OR libya[Text Word] OR libyan arab jamahiriya[Text Word] OR lithuania[Text Word] OR macau[Text Word] OR macao[Text Word] OR macedonia[Text Word] OR madagascar[Text Word] OR malagasy republic[Text Word] OR malawi[Text Word] OR nyasaland[Text Word] OR malaysia[Text Word] OR maldives[Text Word] OR indian ocean[Text Word] OR mali[Text Word] OR malta[Text Word] OR micronesia[Text Word] OR kiribati[Text Word] OR marshall islands[Text Word] OR nauru[Text Word] OR northern mariana islands[Text Word] OR palau[Text Word] OR tuvalu[Text Word] OR mauritania[Text Word] OR mauritius[Text Word] OR mexico[Text Word] OR moldova[Text Word] OR moldovian[Text Word] OR mongolia[Text Word] OR montenegro[Text Word] OR morocco[Text Word] OR ifni[Text Word] OR mozambique[Text Word] OR portuguese east africa[Text Word] OR myanmar[Text Word] OR burma[Text Word] OR namibia[Text Word] OR nepal[Text Word] OR netherlands antilles[Text Word] OR nicaragua[Text Word] OR niger[Text Word] OR nigeria[Text Word] OR oman[Text Word] OR muscat[Text Word] OR pakistan[Text Word] OR panama[Text Word] OR papua new guinea[Text Word] OR paraguay[Text Word] OR peru[Text Word] OR philippines[Text Word] OR philipines[Text Word] OR phillipines[Text Word] OR phillippines[Text Word] OR poland[Text Word] OR polish people's republic[Text Word] OR portugal[Text Word] OR portuguese republic[Text Word] OR puerto rico[Text Word] OR romania[Text Word] OR russia[Text Word] OR russian federation[Text Word] OR ussr[Text Word] OR soviet union[Text Word] OR union of soviet socialist republics[Text Word] OR rwanda[Text Word] OR ruanda[Text Word] OR samoa[Text Word] OR pacific islands[Text Word] OR polynesia[Text Word] OR samoan islands[Text Word] OR sao tome and principe[Text Word] OR saudi arabia[Text Word] OR senegal[Text Word] OR serbia[Text Word] OR seychelles[Text Word] OR sierra leone[Text Word] OR slovakia[Text Word] OR slovak republic[Text Word] OR slovenia[Text Word] OR melanesia[Text Word] OR solomon island[Text Word] OR solomon islands[Text Word] OR norfolk island[Text Word] OR somalia[Text Word] OR south africa[Text Word] OR south sudan[Text Word] OR sri lanka[Text Word] OR ceylon[Text Word] OR saint kitts and nevis[Text Word] OR st kitts and nevis[Text Word] OR saint lucia[Text Word] OR st lucia[Text Word] OR saint vincent[Text Word] OR st vincent[Text Word] OR grenadines[Text Word] OR sudan[Text Word] OR suriname[Text Word] OR surinam[Text Word] OR syria[Text Word] OR syrian arab republic[Text Word] OR tajikistan[Text Word] OR tadjikistan[Text Word] OR tadzhikistan[Text Word] OR tadzhik[Text Word] OR tanzania[Text Word] OR tanganyika[Text Word] OR thailand[Text Word] OR siam[Text Word] OR timor leste[Text Word] OR east timor[Text Word] OR togo[Text Word] OR togolese republic[Text Word] OR tonga[Text Word] OR trinidad[Text Word] OR tobago[Text Word] OR tunisia[Text Word] OR turkey[Text Word] OR turkmenistan[Text Word] OR turkmen[Text Word] OR uganda[Text Word] OR ukraine[Text Word] OR uruguay[Text Word] OR uzbekistan[Text Word] OR uzbek[Text Word] OR vanuatu[Text Word] OR new hebrides[Text Word] OR venezuela[Text Word] OR vietnam[Text Word] OR viet nam[Text Word] OR middle east[Text Word] OR west bank[Text Word] OR gaza[Text Word] OR palestine[Text Word] OR yemen[Text Word] OR yugoslavia[Text Word] OR zambia[Text Word] OR zimbabwe[Text Word] OR northern rhodesia[Text Word] OR global south[Text Word] OR africa south of the sahara[Text Word] OR sub saharan africa[Text Word] OR subsaharan africa[Text Word] OR central africa[Text Word] OR north africa[Text Word] OR northern africa[Text Word] OR magreb[Text Word] OR maghrib[Text Word] OR sahara[Text Word] OR southern africa[Text Word] OR east africa[Text Word] OR eastern africa[Text Word] OR west africa[Text Word] OR western

© 2023 Albarqouni L et al. *JAMA Network Open*.

africa[Text Word] OR west indies[Text Word] OR indian ocean islands[Text Word] OR caribbean[Text Word] OR central america[Text Word] OR latin america[Text Word] OR south america[Text Word] OR central asia[Text Word] OR north asia[Text Word] OR northern asia[Text Word] OR southeastern asia[Text Word] OR south eastern asia[Text Word] OR southeast asia[Text Word] OR south east asia[Text Word] OR western asia[Text Word] OR east europe[Text Word] OR eastern europe[Text Word] OR developing country[Text Word] OR developing countries[Text Word] OR developing nation[Text Word] OR developing nations[Text Word] OR developing population[Text Word] OR developing populations[Text Word] OR developing world[Text Word] OR less developed country[Text Word] OR less developed countries[Text Word] OR less developed nation[Text Word] OR less developed nations[Text Word] OR less developed world[Text Word] OR lesser developed countries[Text Word] OR lesser developed nations[Text Word] OR under developed country[Text Word] OR under developed countries[Text Word] OR under developed nations[Text Word] OR under developed world[Text Word] OR underdeveloped country[Text Word] OR underdeveloped countries[Text Word] OR underdeveloped nation[Text Word] OR underdeveloped nations[Text Word] OR underdeveloped population[Text Word] OR underdeveloped populations[Text Word] OR underdeveloped world[Text Word] OR middle income country[Text Word] OR middle income countries[Text Word] OR middle income nation[Text Word] OR middle income nations[Text Word] OR middle income population[Text Word] OR middle income populations[Text Word] OR low income country[Text Word] OR low income countries[Text Word] OR low income nation[Text Word] OR low income nations[Text Word] OR low income population[Text Word] OR low income populations[Text Word] OR lower income country[Text Word] OR lower income countries[Text Word] OR lower income nations[Text Word] OR lower income population[Text Word] OR lower income populations[Text Word] OR underserved countries[Text Word] OR underserved nations[Text Word] OR underserved population[Text Word] OR underserved populations[Text Word] OR under served population[Text Word] OR under served populations[Text Word] OR deprived countries[Text Word] OR deprived population[Text Word] OR deprived populations[Text Word] OR poor country[Text Word] OR poor countries[Text Word] OR poor nation[Text Word] OR poor nations[Text Word] OR poor population[Text Word] OR poor populations[Text Word] OR poor world[Text Word] OR poorer countries[Text Word] OR poorer nations[Text Word] OR poorer population[Text Word] OR poorer populations[Text Word] OR developing economy[Text Word] OR developing economies[Text Word] OR less developed economy[Text Word] OR less developed economies[Text Word] OR underdeveloped economies[Text Word] OR middle income economy[Text Word] OR middle income economies[Text Word] OR low income economy[Text Word] OR low income economies[Text Word] OR lower income economies[Text Word] OR low gdp[Text Word] OR low gnp[Text Word] OR low gross domestic[Text Word] OR low gross national[Text Word] OR lower gdp[Text Word] OR lower gross domestic[Text Word] OR lmic[Text Word] OR lmics[Text Word] OR third world[Text Word] OR lami country[Text Word] OR lami countries[Text Word] OR transitional country[Text Word] OR transitional countries[Text Word] OR emerging economies[Text Word] OR emerging nation[Text Word] OR emerging nations[Text Word])

Embase
('Medical Overuse'/exp OR Overmedicalization:ti,ab OR Overmedicalisation:ti,ab OR Overtreatment:ti,ab OR Over-treatment:ti,ab OR Overuse:ti,ab OR Unnecessary:ti,ab OR Unwarranted:ti,ab OR Inappropriate:ti,ab OR De-implementation:ti,ab OR Deimplementation:ti,ab OR 'Low-value care':ti,ab OR 'low-value hospital care':ti,ab OR 'low-value healthcare':ti,ab OR 'wasteful care':ti,ab OR 'wasteful healthcare':ti,ab OR 'wasteful hospital care':ti,ab OR 'overuse of healthcare':ti,ab OR 'overuse procedure*':ti,ab OR 'medical overuse':ti,ab OR 'inappropriate

© 2023 Albarqouni L et al. *JAMA Network Open*.

healthcare':ti,ab OR 'inappropriate care':ti,ab OR 'unwanted healthcare':ti,ab OR 'unwanted care':ti,ab OR 'unnecessary healthcare':ti,ab OR 'unnecessary care':ti,ab)

AND

('Surgery'/exp/mj OR "Surgery" OR 'Cesarean Section'/exp/mj OR Arthroplasty/exp/mj OR Arthroscopy/exp/mj OR 'Spine Fusion'/exp/mj OR Surgery:ti,ab OR Surgeries:ti,ab OR Surgical:ti,ab OR Caesarean:ti,ab OR operation:ti,ab OR operative:ti,ab OR operations:ti,ab OR Arthroscopic:ti,ab OR Arthroscopies:ti,ab OR Arthroscopy:ti,ab OR 'carpal tunnel surgery':ti,ab OR 'carpal tunnel release':ti,ab OR 'spinal decompression':ti,ab)

AND

(afghanistan OR albania OR algeria OR "american samoa" OR angola OR antigua OR barbuda OR argentina OR armenia OR armenian OR aruba OR azerbaijan OR bahrain OR bangladesh OR barbados OR belarus OR byelarus OR belorussia OR byelorussian OR belize OR "british honduras" OR benin OR dahomey OR bhutan OR bolivia OR bosnia OR herzegovina OR botswana OR bechuanaland OR brazil OR brasil OR bulgaria OR "burkina faso" OR "burkina fasso" OR "upper volta" OR burundi OR urundi OR "cabo verde" OR "cape verde" OR cambodia OR kampuchea OR "khmer republic" OR cameroon OR cameron OR cameroun OR "central african republic" OR "ubangi shari" OR chad OR chile OR china OR colombia OR comoros OR "comoro islands" OR mayotte OR congo OR zaire OR "costa rica" OR "cote divoire" OR "cote d ivoire" OR "cote divoire" OR "cote d ivoire" OR "ivory coast" OR croatia OR cuba OR cyprus OR "czech republic" OR czechoslovakia OR djibouti OR "french somaliland" OR dominica OR "dominican republic" OR ecuador OR egypt OR "united arab republic" OR "el salvador" OR "equatorial guinea" OR "spanish guinea" OR eritrea OR estonia OR eswatini OR swaziland OR ethiopia OR fiji OR gabon OR "gabonese republic" OR gambia OR georgia OR georgian OR ghana OR "gold coast" OR gibraltar OR greece OR grenada OR guam OR guatemala OR guinea OR guyana OR guiana OR haiti OR hispaniola OR honduras OR hungary OR india OR indonesia OR timor OR iran OR iraq OR "isle of man" OR jamaica OR jordan OR kazakhstan OR kazakh OR kenya OR korea OR kosovo OR kyrgyzstan OR kirghizia OR kirgizstan OR "kyrgyz republic" OR kirghiz OR laos OR "lao pdr" OR "lao peoples democratic republic" OR latvia OR lebanon OR lesotho OR basutoland OR liberia OR libya OR "libyan arab jamahiriya" OR lithuania OR macau OR macao OR macedonia OR madagascar OR "malagasy republic" OR malawi OR nyasaland OR malaysia OR maldives OR "indian ocean" OR mali OR malta OR micronesia OR kiribati OR "marshall islands" OR nauru OR "northern mariana islands" OR palau OR tuvalu OR mauritania OR mauritius OR mexico OR moldova OR moldovian OR mongolia OR montenegro OR morocco OR ifni OR mozambique OR "portuguese east africa" OR myanmar OR burma OR namibia OR nepal OR "netherlands antilles" OR nicaragua OR niger OR nigeria OR oman OR muscat OR pakistan OR panama OR "papua new guinea" OR paraguay OR peru OR philippines OR philipines OR phillipines OR phillippines OR poland OR "polish peoples republic" OR portugal OR "portuguese republic" OR "puerto rico" OR romania OR russia OR "russian federation" OR ussr OR "soviet union" OR "union of soviet socialist republics" OR rwanda OR ruanda OR samoa OR "pacific islands" OR polynesia OR "samoan islands" OR "sao tome" AND principe OR "saudi arabia" OR senegal OR serbia OR seychelles OR "sierra leone" OR slovakia OR "slovak republic" OR slovenia OR melanesia OR "solomon island" OR "solomon islands" OR "norfolk island" OR somalia OR "south africa" OR "south sudan" OR "sri lanka" OR ceylon OR "saint kitts" AND nevis OR "st kitts" AND nevis OR "saint lucia" OR "st lucia" OR "saint vincent" OR "st vincent" OR grenadines OR sudan OR suriname OR surinam OR syria OR "syrian arab republic" OR tajikistan OR tadjikistan OR tadzhikistan OR tadzhik OR tanzania OR tanganyika OR thailand OR siam OR "timor leste" OR "east timor" OR togo OR "togolese republic" OR tonga OR trinidad OR tobago OR tunisia OR turkey OR turkmenistan OR turkmen OR uganda OR ukraine OR uruguay OR uzbekistan OR uzbek OR vanuatu OR "new hebrides" OR venezuela OR vietnam OR "viet

© 2023 Albarqouni L et al. *JAMA Network Open*.

nam" OR "middle east" OR "west bank" OR gaza OR palestine OR yemen OR yugoslavia OR zambia OR zimbabwe OR "northern rhodesia" OR "global south" OR "africa south of the sahara" OR "sub saharan africa" OR "subsaharan africa" OR "central africa" OR "north africa" OR "northern africa" OR magreb OR maghrib OR sahara OR "southern africa" OR "east africa" OR "eastern africa" OR "west africa" OR "western africa" OR "west indies" OR "indian ocean islands" OR caribbean OR "central america" OR "latin america" OR "south america" OR "central asia" OR "north asia" OR "northern asia" OR "southeastern asia" OR "south eastern asia" OR "southeast asia" OR "south east asia" OR "western asia" OR "east europe" OR "eastern europe" OR "developing country" OR "developing countries" OR "developing nation" OR "developing nations" OR "developing population" OR "developing populations" OR "developing world" OR "less developed country" OR "less developed countries" OR "less developed nation" OR "less developed nations" OR "less developed world" OR "lesser developed countries" OR "lesser developed nations" OR "under developed country" OR "under developed countries" OR "under developed nations" OR "under developed world" OR "underdeveloped country" OR "underdeveloped countries" OR "underdeveloped nation" OR "underdeveloped nations" OR "underdeveloped population" OR "underdeveloped populations" OR "underdeveloped world" OR "middle income country" OR "middle income countries" OR "middle income nation" OR "middle income nations" OR "middle income population" OR "middle income populations" OR "low income country" OR "low income countries" OR "low income nation" OR "low income nations" OR "low income population" OR "low income populations" OR "lower income country" OR "lower income countries" OR "lower income nations" OR "lower income population" OR "lower income populations" OR "underserved countries" OR "underserved nations" OR "underserved population" OR "underserved populations" OR "under served population" OR "under served populations" OR "deprived countries" OR "deprived population" OR "deprived populations" OR "poor country" OR "poor countries" OR "poor nation" OR "poor nations" OR "poor population" OR "poor populations" OR "poor world" OR "poorer countries" OR "poorer nations" OR "poorer population" OR "poorer populations" OR "developing economy" OR "developing economies" OR "less developed economy" OR "less developed economies" OR "underdeveloped economies" OR "middle income economy" OR "middle income economies" OR "low income economy" OR "low income economies" OR "lower income economies" OR "low gdp" OR "low gnp" OR "low gross domestic" OR "low gross national" OR "lower gdp" OR "lower gross domestic" OR lmic OR lmics OR "third world" OR "lami country" OR "lami countries" OR "transitional country" OR "transitional countries" OR "emerging economies" OR "emerging nation" OR "emerging nations")

PsycINFO
(Medical Overuse.ti,ab. OR Overmedicalization.ti,ab. OR Overmedicalisation.ti,ab. OR Overtreatment.ti,ab. OR Overtreatment.ti,ab. OR Overuse.ti,ab. OR Unnecessary.ti,ab. OR Unwarranted.ti,ab. OR Inappropriate.ti,ab. OR De-implementation.ti,ab. OR Deimplementation.ti,ab. OR Low-value care.ti,ab. OR low-value hospital care.ti,ab. OR low-value healthcare.ti,ab. OR wasteful care.ti,ab. OR wasteful healthcare.ti,ab. OR wasteful hospital care.ti,ab. OR overuse of healthcare.ti,ab. OR overuse procedure.ti,ab. OR medical overuse.ti,ab. OR inappropriate healthcare.ti,ab. OR inappropriate care.ti,ab. OR unwanted healthcare.ti,ab. OR unwanted care.ti,ab. OR unnecessary healthcare.ti,ab. OR unnecessary care.ti,ab.)

AND

(exp "Surgery"/ OR "Surgery" OR exp "Cesarean Birth"/ OR Surgery.ti,ab. OR Surgeries.ti,ab. OR Surgical.ti,ab. OR Caesarean.ti,ab. OR operation.ti,ab. OR operative.ti,ab. OR operations.ti,ab. OR

© 2023 Albarqouni L et al. *JAMA Network Open*.

Arthroscopic.ti,ab. OR Arthroscopies.ti,ab. OR Arthroscopy.ti,ab. OR "carpal tunnel surgery".ti,ab. OR "carpal tunnel release".ti,ab. OR "spinal decompression".ti,ab. OR "Spinal Fusion".ti,ab.)
AND
(afghanistan.mp. OR albania.mp. OR algeria.mp. OR "american samoa".mp. OR angola.mp. OR antigua.mp. OR barbuda.mp. OR argentina.mp. OR armenia.mp. OR armenian.mp. OR aruba.mp. OR azerbaijan.mp. OR bahrain.mp. OR bangladesh.mp. OR barbados.mp. OR belarus.mp. OR byelarus.mp. OR belorussia.mp. OR byelorussian.mp. OR belize.mp. OR "british honduras".mp. OR benin.mp. OR dahomey.mp. OR bhutan.mp. OR bolivia.mp. OR bosnia.mp. OR herzegovina.mp. OR botswana.mp. OR bechuanaland.mp. OR brazil.mp. OR brasil.mp. OR bulgaria.mp. OR "burkina faso".mp. OR "burkina fasso".mp. OR "upper volta".mp. OR burundi.mp. OR urundi.mp. OR "cabo verde".mp. OR "cape verde".mp. OR cambodia.mp. OR kampuchea.mp. OR "khmer republic".mp. OR cameroon.mp. OR cameron.mp. OR cameroun.mp. OR "central african republic".mp. OR "ubangi shari".mp. OR chad.mp. OR chile.mp. OR china.mp. OR colombia.mp. OR comoros.mp. OR "comoro islands".mp. OR mayotte.mp. OR congo.mp. OR zaire.mp. OR "costa rica".mp. OR "cote d’ivoire".mp. OR "cote d’ ivoire".mp. OR "cote divoire".mp. OR "cote d ivoire".mp. OR "ivory coast".mp. OR croatia.mp. OR cuba.mp. OR cyprus.mp. OR "czech republic".mp. OR czechoslovakia.mp. OR djibouti.mp. OR "french somaliland".mp. OR dominica.mp. OR "dominican republic".mp. OR ecuador.mp. OR egypt.mp. OR "united arab republic".mp. OR "el salvador".mp. OR "equatorial guinea".mp. OR "spanish guinea".mp. OR eritrea.mp. OR estonia.mp. OR eswatini.mp. OR swaziland.mp. OR ethiopia.mp. OR fiji.mp. OR gabon.mp. OR "gabonese republic".mp. OR gambia.mp. OR georgia.mp. OR georgian.mp. OR ghana.mp. OR "gold coast".mp. OR gibraltar.mp. OR greece.mp. OR grenada.mp. OR guam.mp. OR guatemala.mp. OR guinea.mp. OR guyana.mp. OR guiana.mp. OR haiti.mp. OR hispaniola.mp. OR honduras.mp. OR hungary.mp. OR india.mp. OR indonesia.mp. OR timor.mp. OR iran.mp. OR iraq.mp. OR "isle of man".mp. OR jamaica.mp. OR jordan.mp. OR kazakhstan.mp. OR kazakh.mp. OR kenya.mp. OR korea.mp. OR kosovo.mp. OR kyrgyzstan.mp. OR kirghizia.mp. OR kirgizstan.mp. OR "kyrgyz republic".mp. OR kirghiz.mp. OR laos.mp. OR "lao pdr".mp. OR "lao people's democratic republic".mp. OR latvia.mp. OR lebanon.mp. OR lesotho.mp. OR basutoland.mp. OR liberia.mp. OR libya.mp. OR "libyan arab jamahiriya".mp. OR lithuania.mp. OR macau.mp. OR macao.mp. OR macedonia.mp. OR madagascar.mp. OR "malagasy republic".mp. OR malawi.mp. OR nyasaland.mp. OR malaysia.mp. OR maldives.mp. OR "indian ocean".mp. OR mali.mp. OR malta.mp. OR micronesia.mp. OR kiribati.mp. OR "marshall islands".mp. OR nauru.mp. OR "northern mariana islands".mp. OR palau.mp. OR tuvalu.mp. OR mauritania.mp. OR mauritius.mp. OR mexico.mp. OR moldova.mp. OR moldovian.mp. OR mongolia.mp. OR montenegro.mp. OR morocco.mp. OR ifni.mp. OR mozambique.mp. OR "portuguese east africa".mp. OR myanmar.mp. OR burma.mp. OR namibia.mp. OR nepal.mp. OR "netherlands antilles".mp. OR nicaragua.mp. OR niger.mp. OR nigeria.mp. OR oman.mp. OR muscat.mp. OR pakistan.mp. OR panama.mp. OR "papua new guinea".mp. OR paraguay.mp. OR peru.mp. OR philippines.mp. OR philipines.mp. OR phillipines.mp. OR phillippines.mp. OR poland.mp. OR "polish people's republic".mp. OR portugal.mp. OR "portuguese republic".mp. OR "puerto rico".mp. OR romania.mp. OR russia.mp. OR "russian federation".mp. OR ussr.mp. OR "soviet union".mp. OR "union of soviet socialist republics".mp. OR rwanda.mp. OR ruanda.mp. OR samoa.mp. OR "pacific islands".mp. OR polynesia.mp. OR "samoan islands".mp. OR "sao tome" AND principe.mp. OR "saudi arabia".mp. OR senegal.mp. OR serbia.mp. OR seychelles.mp. OR "sierra leone".mp. OR slovakia.mp. OR "slovak republic".mp. OR slovenia.mp. OR melanesia.mp. OR "solomon island".mp. OR "solomon islands".mp. OR "norfolk island".mp. OR somalia.mp. OR "south africa".mp. OR "south sudan".mp. OR "sri lanka".mp. OR ceylon.mp. OR "saint kitts" AND nevis.mp. OR "st kitts" AND nevis.mp. OR "saint lucia".mp. OR "st lucia".mp. OR "saint vincent".mp. OR "st vincent".mp. OR grenadines.mp. OR sudan.mp. OR suriname.mp. OR surinam.mp. OR syria.mp. OR "syrian arab republic".mp. OR tajikistan.mp. OR tadjikistan.mp. OR tadzhikistan.mp. OR tadzhik.mp. OR

© 2023 Albarqouni L et al. *JAMA Network Open*.

tanzania.mp. OR tanganyika.mp. OR thailand.mp. OR siam.mp. OR "timor leste".mp. OR "east timor".mp. OR togo.mp. OR "togolese republic".mp. OR tonga.mp. OR trinidad.mp. OR tobago.mp. OR tunisia.mp. OR turkey.mp. OR turkmenistan.mp. OR turkmen.mp. OR uganda.mp. OR ukraine.mp. OR uruguay.mp. OR uzbekistan.mp. OR uzbek.mp. OR vanuatu.mp. OR "new hebrides".mp. OR venezuela.mp. OR vietnam.mp. OR "viet nam".mp. OR "middle east".mp. OR "west bank".mp. OR gaza.mp. OR palestine.mp. OR yemen.mp. OR yugoslavia.mp. OR zambia.mp. OR zimbabwe.mp. OR "northern rhodesia".mp. OR "global south".mp. OR "africa south of the sahara".mp. OR "sub saharan africa".mp. OR "subsaharan africa".mp. OR "central africa".mp. OR "north africa".mp. OR "northern africa".mp. OR magreb.mp. OR maghrib.mp. OR sahara.mp. OR "southern africa".mp. OR "east africa".mp. OR "eastern africa".mp. OR "west africa".mp. OR "western africa".mp. OR "west indies".mp. OR "indian ocean islands".mp. OR caribbean.mp. OR "central america".mp. OR "latin america".mp. OR "south america".mp. OR "central asia".mp. OR "north asia".mp. OR "northern asia".mp. OR "southeastern asia".mp. OR "south eastern asia".mp. OR "southeast asia".mp. OR "south east asia".mp. OR "western asia".mp. OR "east europe".mp. OR "eastern europe".mp. OR "developing country".mp. OR "developing countries".mp. OR "developing nation".mp. OR "developing nations".mp. OR "developing population".mp. OR "developing populations".mp. OR "developing world".mp. OR "less developed country".mp. OR "less developed countries".mp. OR "less developed nation".mp. OR "less developed nations".mp. OR "less developed world".mp. OR "lesser developed countries".mp. OR "lesser developed nations".mp. OR "under developed country".mp. OR "under developed countries".mp. OR "under developed nations".mp. OR "under developed world".mp. OR "underdeveloped country".mp. OR "underdeveloped countries".mp. OR "underdeveloped nation".mp. OR "underdeveloped nations".mp. OR "underdeveloped population".mp. OR "underdeveloped populations".mp. OR "underdeveloped world".mp. OR "middle income country".mp. OR "middle income countries".mp. OR "middle income nation".mp. OR "middle income nations".mp. OR "middle income population".mp. OR "middle income populations".mp. OR "low income country".mp. OR "low income countries".mp. OR "low income nation".mp. OR "low income nations".mp. OR "low income population".mp. OR "low income populations".mp. OR "lower income country".mp. OR "lower income countries".mp. OR "lower income nations".mp. OR "lower income population".mp. OR "lower income populations".mp. OR "underserved countries".mp. OR "underserved nations".mp. OR "underserved population".mp. OR "underserved populations".mp. OR "under served population".mp. OR "under served populations".mp. OR "deprived countries".mp. OR "deprived population".mp. OR "deprived populations".mp. OR "poor country".mp. OR "poor countries".mp. OR "poor nation".mp. OR "poor nations".mp. OR "poor population".mp. OR "poor populations".mp. OR "poor world".mp. OR "poorer countries".mp. OR "poorer nations".mp. OR "poorer population".mp. OR "poorer populations".mp. OR "developing economy".mp. OR "developing economies".mp. OR "less developed economy".mp. OR "less developed economies".mp. OR "underdeveloped economies".mp. OR "middle income economy".mp. OR "middle income economies".mp. OR "low income economy".mp. OR "low income economies".mp. OR "lower income economies".mp. OR "low gdp".mp. OR "low gnp".mp. OR "low gross domestic".mp. OR "low gross national".mp. OR "lower gdp".mp. OR "lower gross domestic".mp. OR lmic.mp. OR lmics.mp. OR "third world".mp. OR "lami country".mp. OR "lami countries".mp. OR "transitional country".mp. OR "transitional countries".mp. OR "emerging economies".mp. OR "emerging nation".mp. OR "emerging nations".mp.)

Global Index Medicus (WPRIM (Western Pacific); LILACS (Americas); IMSEAR (South-East Asia); IMEMR (Eastern Mediterranean); AIM (Africa))

© 2023 Albarqouni L et al. *JAMA Network Open*.

(tw:("Medical Overuse" OR Overmedicalization OR Overmedicalisation)) OR (ti:(Overtreatment OR Over-treatment OR Overuse OR Unnecessary OR Unwarranted OR Inappropriate OR De-implementation OR Deimplementation))
AND
(tw:("Surgery" OR "Cesarean Section" OR Arthroplasty OR Arthroscopy OR "Spinal Fusion" OR Surgery OR Surgeries OR Surgical OR Caesarean OR operation OR operative OR operations OR Arthroscopic OR Arthroscopies OR Arthroscopy OR "carpal tunnel surgery" OR "carpal tunnel release" OR "spinal decompression"))

© 2023 Albarqouni L et al. *JAMA Network Open*.

eTable. Characteristics of included studies in the scoping review (n=133).

Author, year	Country, Income level, WHO country category	Study design (analysis approach)	Sample size (P: Surgical procedures, I: Individuals)	Condition; Emergency/ Elective; Major/ Minor	Main context and themes
**Huang K et al., 2013**[1]	Single (China, East Asia & Pacific, Upper middle income)	Observational, e.g. cohort or cross-sectional studies (Mixed)	P (1275); I (2326)	Maternal; Unclear; Major	Drivers and factors related to overuse of medications and
**Kale I, 2021**[2]	Single (Turkey, Europe & Central Asia, Upper middle income)	Observational, e.g. cohort or cross-sectional studies (Quantitative)	P (13077); I (32338)	Maternal; Emergency; Major	Drivers and factors related to overuse of medications and
**Khresheh R et al., 2020**[3]	Single (Jordan, Middle East & North Africa, Upper middle income)	Observational, e.g. cohort or cross-sectional studies (Quantitative)	P (); I (112)	Maternal; Emergency; Minor	Drivers and factors related to overuse of medications and
**Liao Z et al., 2019**[4]	Single (China, East Asia & Pacific, Upper middle income)	Observational, e.g. cohort or cross-sectional studies (Quantitative)	P (18468); I (27977)	Maternal; Unclear; Major	Drivers and factors related to overuse of medications and
**Long Q et al., 2022**[5]	Single (China, East Asia & Pacific, Upper middle income)	Observational, e.g. cohort or cross-sectional studies (Quantitative)	P (9973); I (23053)	Maternal; Unclear; Major	Drivers and factors related to overuse of medications and
**Mandong BM et al., 2005**[6]	Single (Nigeria, Sub-Saharan Africa, Lower middle income)	Observational, e.g. cohort or cross-sectional studies (Quantitative)	P (87); I (87)	GU; Elective; Major	Drivers and factors related to overuse of medications and
**Maurya PB et al., 2015**[7]	Single (India, South Asia, Lower middle income)	Observational, e.g. cohort or cross-sectional studies (Quantitative)	P (128); I (128)	Cancer; Elective; Major	Drivers and factors related to overuse of medications and
**Wang E, 2017**[8]	Single (China, East Asia & Pacific, Upper middle income)	Observational, e.g. cohort or cross-sectional studies (Qualitative)	P (26); I (26)	Maternal; Unclear; Major	Drivers and factors related to overuse of medications and
**Rao C et al., 2015**[9]	Single (China, East Asia & Pacific, Upper middle income)	Observational, e.g. cohort or cross-sectional studies (Quantitative)	P (2535); I (3186)	CVS; Emergency; Major	Consequence of overuse of medications and

© 2023 Albarqouni L et al. *JAMA Network Open*.

**Abdel-Aleem H et al., 2017[10]**	Single (Egypt, Arab Rep., Middle East & North Africa, Lower middle income)	Observational, e.g. cohort or cross-sectional studies (Quantitative)	P (1); I (1000)	Maternal; Elective; Major	Extent or estimates and Drivers and factors related to overuse of medications
**Aminu M et al., 2014[11]**	Single (Bangladesh, South Asia, Lower middle income)	Observational, e.g. cohort or cross-sectional studies (Mixed)	P (1); I (530)	Maternal; Mixed; Major	Drivers and factors related to overuse of medications and
**Bakker W et al., 2021[12]**	Single (Malawi, Sub-Saharan Africa, Low income)	Observational, e.g. cohort or cross-sectional studies (Mixed)	P (1); I (3239)	Maternal; Mixed; Major	Drivers and factors related to overuse of medications and
**Beyene MG et al., 2021[13]**	Single (Ethiopia, Sub-Saharan Africa, Low income)	Observational, e.g. cohort or cross-sectional studies (Quantitative)	P (1); I (92273)	Maternal; Mixed; Major	Drivers and factors related to overuse of medications and
**Deng R et al., 2021[14]**	Single (China, East Asia & Pacific, Upper middle income)	Observational, e.g. cohort or cross-sectional studies (Quantitative)	P (462); I (1283)	Maternal; Mixed; Major	Drivers and factors related to overuse of medications and
**Estellita Lins F et al., 1981[15]**	Single (Brazil, Latin America & Caribbean, Upper middle income)	Observational, e.g. cohort or cross-sectional studies (Quantitative)	P (499); I (1819)	Maternal; Mixed; Major	Drivers and factors related to overuse of medications and Extent or estimates
**Feng XL et al., 2014[16]**	Single (China, East Asia & Pacific, Upper middle income)	Review (Qualitative)	P (); I (82)	Maternal; Mixed; Major	Extent or estimates and Drivers and factors related to overuse of medications
**França GV et al., 2016[17]**	Single (Brazil, Latin America & Caribbean, Upper middle income)	Observational, e.g. cohort or cross-sectional studies (Mixed)	P (); I ()	Maternal; Mixed; Major	Extent or estimates and Drivers and factors related to overuse of medications
**Gu N et al., 2021[18]**	Single (China, East Asia & Pacific, Upper middle income)	Observational, e.g. cohort or cross-sectional studies (Mixed)	P (22676); I (52449)	Maternal; Mixed; Major	Drivers and factors related to overuse of medications and Extent or estimates
**Haider MR et al., 2018[19]**	Single (Bangladesh, South Asia, Lower middle income)	Observational, e.g. cohort or cross-sectional studies (Quantitative)	P (3323); I (18733)	Maternal; Mixed; Major	Consequence of overuse of medications and
**Hassan EMA, 2021[20]**	Single (Egypt, Arab Rep., Middle East & North Africa, Lower middle income)	Observational, e.g. cohort or cross-sectional studies (Quantitative)	P (1479); I (2333)	Maternal; Mixed; Major	Extent or estimates and Drivers and factors related to overuse of medications

© 2023 Albarqouni L et al. *JAMA Network Open*.

**Howes R et al., 2017**[21]	Single (Afghanistan, South Asia, Low income)	Observational, e.g. cohort or cross-sectional studies (Qualitative)	P (); I (180)	GIT; Emergency; Major	Drivers and factors related to overuse of medications and
**Hoxha I et al., 2019**[22]	Single (Kosovo, Europe & Central Asia, Upper middle income)	Observational, e.g. cohort or cross-sectional studies (Quantitative)	P (178); I (859)	Maternal; Mixed; Major	Drivers and factors related to overuse of medications and
**Kouanda S et al., 2014**[23]	Single (Burkina Faso, Sub-Saharan Africa, Low income)	Observational, e.g. cohort or cross-sectional studies (Mixed)	P (); I (300)	Maternal; Mixed; Major	Extent or estimates and Drivers and factors related to overuse of medications
**Leone T, 2014**[24]	Single (India, South Asia, Lower middle income)	Observational, e.g. cohort or cross-sectional studies (Mixed)	P (); I ()	Maternal; Mixed; Major	Drivers and factors related to overuse of medications and
**Liang J et al., 2018**[25]	Single (China, East Asia & Pacific, Upper middle income)	Observational, e.g. cohort or cross-sectional studies (Mixed)	P (); I (3078101)	Maternal; Mixed; Major	Drivers and factors related to overuse of medications and
**Long Q et al., 2012**[26]	Single (China, East Asia & Pacific, Upper middle income)	Observational, e.g. cohort or cross-sectional studies (Quantitative)	P (); I (3550)	Maternal; Mixed; Major	Drivers and factors related to overuse of medications and
**Majlesi M et al., 2020**[27]	Single (Iran, Islamic Rep., Middle East & North Africa, Upper middle income)	Observational, e.g. cohort or cross-sectional studies (Mixed)	P (); I (466)	Maternal; Mixed; Major	Potential Solutions and
**Mohammadi S et al., 2018**[28]	Single (Iran, Islamic Rep., Middle East & North Africa, Upper middle income)	Observational, e.g. cohort or cross-sectional studies (Mixed)	P (8513); I (13169)	Maternal; Mixed; Major	Extent or estimates and Drivers and factors related to overuse of medications
**Nelson JP, 2017**[29]	Single (Uganda, Sub-Saharan Africa, Low income)	Observational, e.g. cohort or cross-sectional studies (Mixed)	P (); I (200)	Maternal; Mixed; Major	Drivers and factors related to overuse of medications and
**Elnakib S et al., 2019**[30]	Single (Egypt, Arab Rep., Middle East & North Africa, Lower middle income)	Observational, e.g. cohort or cross-sectional studies (Mixed)	P (4357); I (275)	Maternal; Mixed; Major	Drivers and factors related to overuse of medications and Extent or estimates
**Ganji F et al., 2006**[31]	Single (Iran, Islamic Rep., Middle East & North Africa, Upper middle income)	Interventional, e.g. randomised trial (Mixed)	P (); I (171)	Maternal; Mixed; Major	Potential Solutions and

© 2023 Albarqouni L et al. *JAMA Network Open*.

**Gonzalez-Perez GJ et al., 2001[32]**	Single (Mexico, Latin America & Caribbean, Upper middle income)	Observational, e.g. cohort or cross-sectional studies (Quantitative)	P (); I ()	Maternal; Mixed; Major	Extent or estimates and
**Hakimi S et al., 2020[33]**	Single (Iran, Islamic Rep., Middle East & North Africa, Upper middle income)	Observational, e.g. cohort or cross-sectional studies (Quantitative)	P (); I ()	Maternal; Mixed; Major	Potential Solutions and
**Hatamleh R et al., 2019[34]**	Single (Jordan, Middle East & North Africa, Upper middle income)	Others (Qualitative)	P (); I (35)	Maternal; Elective; Major	Drivers and factors related to overuse of medications and
**Hoxha I et al., 2021[35]**	#N/A	Observational, e.g. cohort or cross-sectional studies (Quantitative)	P (); I ()	Maternal; Mixed; Major	Drivers and factors related to overuse of medications and Extent or estimates
**Hu Y et al., 2016[36]**	Single (China, East Asia & Pacific, Upper middle income)	Observational, e.g. cohort or cross-sectional studies (Quantitative)	P (33476); I ()	Maternal; Elective; Major	Consequence of overuse of medications and Extent or estimates
**Ion RC et al., 2013[37]**	Single (Uganda, Sub-Saharan Africa, Low income)	Interventional, e.g. randomised trial (Quantitative)	P (); I (2012)	Maternal; Unclear; Major	Potential Solutions and
**Maaløe N et al., 2012[38]**	Single (Tanzania, Sub-Saharan Africa, Lower middle income)	Observational, e.g. cohort or cross-sectional studies (Quantitative)	P (); I (400)	Maternal; Emergency; Major	Extent or estimates and
**Perrotta C et al., 2022[39]**	Single (Argentina, Latin America & Caribbean, Upper middle income)	Observational, e.g. cohort or cross-sectional studies (Mixed)	P (.); I (1081)	Maternal; Mixed; Major	Drivers and factors related to overuse of medications and
**Schantz C et al., 2020[40]**	#N/A	Others (Qualitative)	P (); I (25)	Maternal; Mixed; Major	Drivers and factors related to overuse of medications and
**Sriussadaporn S et al., 1993[41]**	Single (Thailand, East Asia & Pacific, Upper middle income)	Observational, e.g. cohort or cross-sectional studies (Quantitative)	P (); I (279)	Trauma; Emergency; Major	Consequence of overuse of medications and Extent or estimates
**Su Y et al., 2020[42]**	Single (China, East Asia & Pacific, Upper middle income)	Interventional, e.g. randomised trial (Quantitative)	P (); I (4629)	Maternal; Elective; Major	Potential Solutions and Drivers and factors related to overuse of medications

© 2023 Albarqouni L et al. *JAMA Network Open*.

**Tang Y et al., 2021**[43]	Single (China, East Asia & Pacific, Upper middle income)	Observational, e.g. cohort or cross-sectional studies (Quantitative)	P (); I (4778)	Maternal; Mixed; Major	Consequence of overuse of medications and Extent or estimates
**Wang X et al., 2017**[44]	Single (China, East Asia & Pacific, Upper middle income)	Observational, e.g. cohort or cross-sectional studies (Quantitative)	P (61084); I (112138)	Maternal; Mixed; Major	Extent or estimates and
**Yang J et al., 2021**[45]	Single (China, East Asia & Pacific, Upper middle income)	Observational, e.g. cohort or cross-sectional studies (Quantitative)	P (); I (900)	Maternal; Elective; Major	Drivers and factors related to overuse of medications and
**Zarifsanaiey N et al., 2020**[46]	Single (Iran, Islamic Rep., Middle East & North Africa, Upper middle income)	Interventional, e.g. randomised trial (Quantitative)	P (); I (130)	Maternal; Elective; Major	Potential Solutions and
**Kaboré C et al., 2019**[47]	Single (Burkina Faso, Sub-Saharan Africa, Low income)	Interventional, e.g. randomised trial (Quantitative)	P (); I (4174)	Maternal; Elective; Major	Potential Solutions and
**Litorp H et al., 2013**[48]	Single (Tanzania, Sub-Saharan Africa, Lower middle income)	Others (Quantitative)	P (); I (137094)	Maternal; Mixed; Major	Extent or estimates and
**Muñoz-Enciso JM et al., 2011**[49]	Single (Mexico, Latin America & Caribbean, Upper middle income)	Observational, e.g. cohort or cross-sectional studies (Mixed)	P (); I ()	Maternal; Mixed; Major	Drivers and factors related to overuse of medications and
**Peiris CJ, 2010**[50]	#N/A	Observational, e.g. cohort or cross-sectional studies (Mixed)	P (); I ()	CVS; ; Minor	Extent or estimates and
**Smith-Oka V, 2022**[51]	Single (Mexico, Latin America & Caribbean, Upper middle income)	Observational, e.g. cohort or cross-sectional studies (Mixed)	P (); I (12)	Maternal; Unclear; Major	Drivers and factors related to overuse of medications and
**Zarshenas M et al., 2020**[52]	Multiple (Iran, Islamic Rep., Middle East & North Africa, Upper middle income)	Observational, e.g. cohort or cross-sectional studies (Quantitative)	P (); I (700)	Maternal; Mixed; Major	Drivers and factors related to overuse of medications and
**Zhang L et al., 2020**[53]	Single (China, East Asia & Pacific, Upper middle income)	Interventional, e.g. randomised trial (Quantitative)	P (); I (11273)	Maternal; Mixed; Major	Potential Solutions and

© 2023 Albarqouni L et al. *JAMA Network Open*.

**Ahmed HO et al., 2020**[54]	Single (Iraq, Middle East & North Africa, Upper middle income)	Observational, e.g. cohort or cross-sectional studies (Quantitative)	P (); I (5847)	GIT; Emergency; Major	Drivers and factors related to overuse of medications and
**Ahmed MS et al., 2022**[55]	Single (Bangladesh, South Asia, Lower middle income)	Observational, e.g. cohort or cross-sectional studies (Quantitative)	P (); I (4903)	Maternal; Mixed; Major	Drivers and factors related to overuse of medications and
**da Matta Machado Fernandes L et al., 2021**[56]	Single (Brazil, Latin America & Caribbean, Upper middle income)	Observational, e.g. cohort or cross-sectional studies (Mixed)	P (); I (555)	Maternal; Unclear; Major	Drivers and factors related to overuse of medications and Potential Solutions
**Darsareh F et al., 2019**[57]	Single (Iran, Islamic Rep., Middle East & North Africa, Upper middle income)	Observational, e.g. cohort or cross-sectional studies (Quantitative)	P (); I (350)	Maternal; Elective; Major	Potential Solutions and
**Desai S et al., 2011**[58]	Single (India, South Asia, Lower middle income)	Observational, e.g. cohort or cross-sectional studies (Quantitative)	P (264); I (2010)	Maternal; Mixed; Major	Drivers and factors related to overuse of medications and Extent or estimates
**Dhakal Rai S et al., 2019**[59]	Single (Nepal, South Asia, Lower middle income)	Observational, e.g. cohort or cross-sectional studies (Quantitative)	P (); I ()	Maternal; Mixed; Major	Extent or estimates and
**Karthikeyan G et al., 2017**[60]	Single (India, South Asia, Lower middle income)	Interventional, e.g. randomised trial (Quantitative)	P (89787); I ()	CVS; Elective; Major	Potential Solutions and
**Khan RN et al., 2018**[61]	Single (Pakistan, South Asia, Lower middle income)	Observational, e.g. cohort or cross-sectional studies (Quantitative)	P (); I (210)	Cancer; Elective; Major	Potential Solutions and
**Liabsuetrakul T et al., 2019**[62]	Single (Thailand, East Asia & Pacific, Upper middle income)	Observational, e.g. cohort or cross-sectional studies (Quantitative)	P (); I (N/A)	Maternal; Mixed; Major	Consequence of overuse of medications and
**Litorp H et al., 2015**[63]	Single (Tanzania, Sub-Saharan Africa, Lower middle income)	Others (Qualitative)	P (); I (32)	Maternal; Unclear; Major	Drivers and factors related to overuse of medications and Extent or estimates

© 2023 Albarqouni L et al. *JAMA Network Open*.

**Belizán JM et al., 1999[64]**	#N/A	Observational, e.g. cohort or cross-sectional studies (Qualitative)	P (2178714); I ()	Maternal; Mixed; Major	Extent or estimates and
**Gedefaw G et al., 2021[65]**	Single (Ethiopia, Sub-Saharan Africa, Low income)	Observational, e.g. cohort or cross-sectional studies (Quantitative)	P (); I (4757)	Maternal; Unclear; Major	Drivers and factors related to overuse of medications and Extent or estimates
**Giang HTN et al., 2018[66]**	Single (Vietnam, East Asia & Pacific, Lower middle income)	Observational, e.g. cohort or cross-sectional studies (Quantitative)	P (); I (20535)	Maternal; Mixed; Major	Extent or estimates and Drivers and factors related to overuse of medications
**Islam MT et al., 2015[67]**	Single (Bangladesh, South Asia, Lower middle income)	Observational, e.g. cohort or cross-sectional studies (Quantitative)	P (1075); I (3329)	Maternal; Emergency; Major	Extent or estimates and Drivers and factors related to overuse of medications
**Kaboré C et al., 2016[68]**	Single (Burkina Faso, Sub-Saharan Africa, Low income)	Observational, e.g. cohort or cross-sectional studies (Quantitative)	P (2138); I ()	Maternal; Unclear; Major	Extent or estimates and Drivers and factors related to overuse of medications
**Lin S et al., 2020[69]**	Single (China, East Asia & Pacific, Upper middle income)	Observational, e.g. cohort or cross-sectional studies (Quantitative)	P (3980); I ()	CVS; Mixed; Major	Extent or estimates and Drivers and factors related to overuse of medications
**Mumtaz S et al., 2017[70]**	single (Pakistan, South Asia, Lower middle income)	Observational, e.g. cohort or cross-sectional studies (Quantitative)	P (); I (30192)	Maternal; Unclear; Major	Extent or estimates and Drivers and factors related to overuse of medications
**Mumtaz Z et al., 2020[71]**	single (pakistan, South Asia, Lower middle income)	Others (Qualitative)	P (); I (318)	Maternal; Unclear; Major	Drivers and factors related to overuse of medications and Extent or estimates
**Ostovar R et al., 2012[72]**	single (Iran, Islamic Rep., Middle East & North Africa, Upper middle income)	Observational, e.g. cohort or cross-sectional studies (Quantitative)	P (); I (10)	Maternal; Unclear; Major	Potential Solutions and
**Patil D et al., 2017[73]**	single (india, South Asia, Lower middle income)	Observational, e.g. cohort or cross-sectional studies (Quantitative)	P (894); I (857)	CVS; Elective; Major	Extent or estimates and Potential Solutions
**Rafiq MS et al., 2015[74]**	single (pakistan, South Asia, Lower middle income)	Observational, e.g. cohort or cross-sectional studies (Quantitative)	P (); I (408)	GIT; Emergency; Major	Potential Solutions and

© 2023 Albarqouni L et al. *JAMA Network Open*.

**Santas G et al., 2018**[75]	single (Turkey, Europe & Central Asia, Upper middle income)	Observational, e.g. cohort or cross-sectional studies (Quantitative)	P (); I ()	Maternal; Unclear; Major	Extent or estimates and Drivers and factors related to overuse of medications
**Srisomboon J et al., 2000**[76]	single (Thailand, East Asia & Pacific, Upper middle income)	Observational, e.g. cohort or cross-sectional studies (Quantitative)	P (70); I (70)	Maternal; Mixed; Major	Drivers and factors related to overuse of medications and
**van den Bergh JE et al., 2003**[77]	single (Zimbabwe, Sub-Saharan Africa, Lower middle income)	Observational, e.g. cohort or cross-sectional studies (Quantitative)	P (965); I (3589)	Maternal; Unclear; Minor	Extent or estimates and Drivers and factors related to overuse of medications
**Zamboni K et al., 2019**[78]	#N/A	Review (Qualitative)	P (); I ()	Maternal; Mixed; Major	Potential Solutions and
**Ahmmed F et al., 2021**[79]	Single (Bangladesh, South Asia, Lower middle income)	Observational, e.g. cohort or cross-sectional studies (Quantitative)	P (1065); I (4433)	Maternal; Mixed; Major	Drivers and factors related to overuse of medications and
**Al Rifai R, 2014**[80]	Single (Jordan, Middle East & North Africa, Upper middle income)	Observational, e.g. cohort or cross-sectional studies (Quantitative)	P (16774); I (28234)	Maternal; Mixed; Major	Drivers and factors related to overuse of medications and
**Boatin AA et al., 2018**[81]	#VALUE!	Observational, e.g. cohort or cross-sectional studies (Quantitative)	P (); I ()	Maternal; Mixed; Major	Extent or estimates and
**Cheng K et al., 2019**[82]	Single (China, East Asia & Pacific, Upper middle income)	Observational, e.g. cohort or cross-sectional studies (Quantitative)	P (246); I ()	Respiratory; Elective; Major	Extent or estimates and
**Phadungkiatwattana P et al., 2011**[83]	Single (Thailand, East Asia & Pacific, Upper middle income)	Observational, e.g. cohort or cross-sectional studies (Quantitative)	P (2841); I (2841)	Maternal; Mixed; Major	Drivers and factors related to overuse of medications and
**Ribeiro LB, 2016**[84]	Single (Brazil, Latin America & Caribbean, Upper middle income)	Observational, e.g. cohort or cross-sectional studies (Quantitative)	P (744); I (744)	Maternal; Unclear; Major	Extent or estimates and Drivers and factors related to overuse of medications

© 2023 Albarqouni L et al. *JAMA Network Open*.

**Rudey EL et al., 2020[85]**	Single (Brazil, Latin America & Caribbean, Upper middle income)	Observational, e.g. cohort or cross-sectional studies (Quantitative)	P (6580432); I (6580432)	Maternal; Unclear; Major	Extent or estimates and Drivers and factors related to overuse of medications
**Saleh Gargari S et al., 2019[86]**	Single (Iran, Islamic Rep., Middle East & North Africa, Upper middle income)	Observational, e.g. cohort or cross-sectional studies (Quantitative)	P (61); I (61)	Maternal; Unclear; Major	Consequence of overuse of medications and Extent or estimates
**Shirzad M et al., 2021[87]**	Single (Iran, Islamic Rep., Middle East & North Africa, Upper middle income)	Review (Mixed)	P (); I (4814)	Maternal; Unclear; Major	Drivers and factors related to overuse of medications and
**Singh P et al., 2018[88]**	Single (India, South Asia, Lower middle income)	Observational, e.g. cohort or cross-sectional studies (Quantitative)	P (4160); I (22111)	Maternal; Mixed; Major	Drivers and factors related to overuse of medications and
**Torloni MR et al., 2020[89]**	#N/A	Review (Quantitative)	P (); I (466)	Maternal; Mixed; Major	Potential Solutions and
**Türkmen A et al., 2016[90]**	Single (Turkey, Europe & Central Asia, Upper middle income)	Observational, e.g. cohort or cross-sectional studies (Quantitative)	P (3); I (37)	Trauma; ; Major	Consequence of overuse of medications and
**Takegata M et al., 2020[91]**	Single (Vietnam, East Asia & Pacific, Lower middle income)	Others (Qualitative)	P (); I (48)	Maternal; Mixed; Major	Drivers and factors related to overuse of medications and
**Tenge RK et al., 1999[92]**	Single (Kenya, Sub-Saharan Africa, Lower middle income)	Observational, e.g. cohort or cross-sectional studies (Quantitative)	P (33); I (55)	Trauma; Emergency; Major	Extent or estimates and
**Ostovar R et al., 2010[93]**	Single (Iran, Islamic Rep., Middle East & North Africa, Upper middle income)	Others (Quantitative)	P (294); I (294)	Maternal; Mixed; Major	Extent or estimates and
**Rahman MM et al., 2018[94]**	Single (Bangladesh, South Asia, Lower middle income)	Observational, e.g. cohort or cross-sectional studies (Quantitative)	P (1122); I (4627)	Maternal; Mixed; Major	Drivers and factors related to overuse of medications and

© 2023 Albarqouni L et al. *JAMA Network Open*.

**Rubagumya F et al., 2020**[95]	#N/A	Others (Mixed)	P (); I ()	Maternal; Unclear; Major	Potential Solutions and
**Colomar M et al., 2014**[96]	Single (Nicaragua, Latin America & Caribbean, Lower middle income)	Others (Qualitative)	P (); I (17)	Maternal; Mixed; Major	Drivers and factors related to overuse of medications and Potential Solutions
**Colomar M et al., 2021**[97]	#N/A	Review (Qualitative)	P (); I ()	Maternal; Unclear; Major	Drivers and factors related to overuse of medications and
**Dankwah E et al., 2019**[98]	Single (Ghana, Sub-Saharan Africa, Lower middle income)	Observational, e.g. cohort or cross-sectional studies (Quantitative)	P (11835); I (4229)	Maternal; Mixed; Major	Drivers and factors related to overuse of medications and
**Fernandes LMM et al., 2020**[99]	Single (Brazil, Latin America & Caribbean, Upper middle income)	Observational, e.g., cohort or cross-sectional studies (Quantitative)	P (); I (1287)	Maternal; Mixed; Major	Potential Solutions and
**Ghotbi Fa, 2014**[100]	Single (Iran, Islamic Rep., Middle East & North Africa, Upper middle income)	Observational, e.g., cohort or cross-sectional studies (Quantitative)	P (501); I (600)	Maternal; Elective; Major	Drivers and factors related to overuse of medications and Extent or estimates
**Ola ER et al., 2002**[101]	Single (Nigeria, Sub-Saharan Africa, Lower middle income)	Observational, e.g., cohort or cross-sectional studies (Quantitative)	P (); I (1007)	Maternal; Unclear; Minor	Drivers and factors related to overuse of medications and Extent or estimates
**Shi Y et al., 2016**[102]	Single (China, East Asia & Pacific, Upper middle income)	Observational e.g., cohort or cross-sectional studies (Quantitative)	P (); I (977)	Maternal; Unclear; Major	Drivers and factors related to overuse of medications and
**Wang L et al., 2016**[103]	Single (China, East Asia & Pacific, Upper middle income)	Observational, e.g., cohort or cross-sectional studies (Quantitative)	P (); I (2345)	Maternal; Mixed; Major	Drivers and factors related to overuse of medications and Extent or estimates
**Zimmo K et al., 2018**[104]	Single (West Bank and Gaza, Middle East & North Africa, Lower middle income)	Observational, e.g., cohort or cross-sectional studies (Quantitative)	P (); I (29165)	Maternal; Unclear; Major	Drivers and factors related to overuse of medications and Extent or estimates
**Prado DS et al., 2017**[105]	Single (Brazil, Latin America & Caribbean, Upper middle income)	Observational, e.g. cohort or cross-sectional studies (Quantitative)	P (311); I (768)	Maternal; Unclear; Major	Drivers and factors related to overuse of medications and

© 2023 Albarqouni L et al. *JAMA Network Open*.

**Roy N et al., 2021[106]**	Single (India, South Asia, Lower middle income)	Review (Quantitative)	P (); I ()	Maternal; Unclear; Major	Drivers and factors related to overuse of medications and
**Schantz C et al., 2018[107]**	Multiple	Observational, e.g. cohort or cross-sectional studies (Quantitative)	P (4566); I (4566)	Maternal; Unclear; Major	Extent or estimates and Drivers and factors related to overuse of medications
**Shirzad M et al., 2019[108]**	Single (Iran, Islamic Rep., Middle East & North Africa, Upper middle income)	Others (Qualitative)	P (15); I (26)	Maternal; Unclear; Major	Drivers and factors related to overuse of medications and
**Suwanrath C et al, 2021[109]**	Single (Thailand, East Asia & Pacific, Upper middle income)	Others (Qualitative)	P (); I (27)	Maternal; Unclear; Major	Drivers and factors related to overuse of medications and
**Taviloglu K et al, 1998[110]**	Single (Turkey, Europe & Central Asia, Upper middle income)	Observational e.g. cohort or cross-sectional studies (Quantitative)	P (58); I (200)	Trauma; Emergency; Major	Potential Solutions and
**Dias MA et al, 2016[111]**	Single (Brazil, Latin America & Caribbean, Upper middle income)	Observational e.g. cohort or cross-sectional studies (Mixed)	P (4079); I (23894)	Maternal; Mixed; Major	Extent or estimates and
**Galvao R et al, 2018[112]**	Single (Brazil, Latin America & Caribbean, Upper middle income)	Observational e.g. cohort or cross-sectional studies (Qualitative)	P (18); I (19)	Maternal; Elective; Major	Potential Solutions and
**Gmez OL et al, 1999[113]**	Single (Colombia, Latin America & Caribbean, Upper middle income)	Observational e.g. cohort or cross-sectional studies (Mixed)	P (229); I (416)	Maternal; Mixed; Major	Extent or estimates and
**Kunthonkitidej K et al, 2001[114]**	Single (Thailand, East Asia & Pacific, Upper middle income)	Observational e.g. cohort or cross-sectional studies (Quantitative)	P (); I (9887)	Maternal; Unclear; Major	Potential Solutions and
**Lansky S et al, 2018[115]**	Single (Brazil, Latin America & Caribbean, Upper middle income)	Observational e.g. cohort or cross-sectional studies (Quantitative)	P (255); I (22621)	Maternal; Unclear; Major	Potential Solutions and

© 2023 Albarqouni L et al. *JAMA Network Open*.

**Lukas E et al, 2020[116]**	Single (Germany, Europe & Central Asia, High income)	Observational e.g. cohort or cross-sectional studies (Mixed)	P (3); I (7)	GIT; Elective; Major	Extent or estimates and
**Misaeli C et al, 2017[117]**	Single (Tanzania, Sub-Saharan Africa, Lower middle income)	Observational e.g. cohort or cross-sectional studies (Qualitative)	P (106); I (440)	Maternal; Elective; Major	Drivers and factors related to overuse of medications and
**Dekker L et al, 2018[118]**	Single (Tanzania, Sub-Saharan Africa, Lower middle income)	Observational e.g. cohort or cross-sectional studies (Quantitative)	P (); I (1868)	Maternal; Emergency; Major	Extent or estimates and Potential Solutions
**Ajeet S et al, 2013[119]**	Single (India, South Asia, Lower middle income)	Observational e.g. cohort or cross-sectional studies ()	P (); I (272)	Maternal; Mixed; Major	Extent or estimates and Drivers and factors related to overuse of medications
**Ahmed MS et al, 2022[120]**	Single (Bangladesh, South Asia, Lower middle income)	Observational e.g. cohort or cross-sectional studies (Quantitative)	P (); I (4903)	Maternal; Mixed; Major	Extent or estimates and Drivers and factors related to overuse of medications
**Albertal M et al, 2010[121]**	Single (Argentina, Latin America & Caribbean, Upper middle income)	Observational e.g. cohort or cross-sectional studies (Quantitative)	P (568); I ()	Cardiac; ; Major	Extent or estimates and
**Alcantara LLM et al, 2020[122]**	Single (Brazil, Latin America & Caribbean, Upper middle income)	Observational e.g. cohort or cross-sectional studies (Qualitative)	P (); I (456089)	Maternal; Mixed; Major	Extent or estimates and Drivers and factors related to overuse of medications
**Al Rifai R, 2014[123]**	Single (Egypt, Middle East & North Africa, Upper middle income)	Observational e.g. cohort or cross-sectional studies (Quantitative)	P (); I (29000)	Maternal; Mixed; Major	Extent or estimates and Drivers and factors related to overuse of medications
**Ali Y et al, 2018[124]**	Single (Pakistan, South Asia, Lower middle income)	Observational e.g. cohort or cross-sectional studies (Quantitative)	P (); I (	Maternal; Mixed; Major	Drivers and factors related to overuse of medications and Potential Solutions
**Althabe F et al, 2002[125]**		Observational e.g. cohort or cross-sectional studies (Quantitative)	P (); I (94472)	Maternal; Emergency; Minor	Extent or estimates and consequence of overuse of medications
**Althabe F et al, 2004[126]**		Interventional e.g. randomised trial (Quantitative)	P (); I (149276)	Maternal; Elective; Major	Potential Solutions and Potential Solutions

© 2023 Albarqouni L et al. *JAMA Network Open*.

**Alzate MM et al, 2019**[127]	Single (Colombia, Latin America & Caribbean, Upper middle income)	Observational e.g. cohort or cross-sectional studies (Quantitative)	P (); I (9977)	Maternal; Mixed; Major	Drivers and factors related to overuse of medications and
**Bakker W et al, 2020**[128]	Single (Malawi, Sub-Saharan Africa, Low income)	Interventional e.g. randomised trial (Quantitative)	P (); I (645)	Maternal; Mixed; Major	Potential Solutions and
**Betran AP et al, 2021**[129]		Observational e.g. cohort or cross-sectional studies (Quantitative)	P (); I ()	Maternal; Mixed; Major	Extent or estimates and Drivers and factors related to overuse of medications
**Bhatia M et al, 2020**[130]	Single (India, South Asia, Lower middle income)	Observational e.g., cohort or cross-sectional studies (Quantitative)	P (); I (190898)	Maternal; Mixed; Major	Drivers and factors related to overuse of medications and
**Campero L et al, 2007**[131]	Single (Mexico, Latin America & Caribbean, Upper middle income)	Observational e.g., cohort or cross-sectional studies (Quantitative)	P (992); I (847)	Maternal; Mixed; Major	Extent or estimates and
**Chanrachakul B et al, 2000**[132]	Single (Thailand, East Asia & Pacific, Upper middle income)	Observational e.g., cohort or cross-sectional studies (Quantitative)	P (); I ()	Maternal; Mixed; Major	Extent or estimates and
**Cheng YM et al, 2003**[133]	Single (China, East Asia & Pacific, Upper middle income)	Observational e.g., cohort or cross-sectional studies (Quantitative)	P (); I (14071)	Maternal; Mixed; Major	Extent or estimates and Drivers and factors related to overuse of medications

© 2023 Albarqouni L et al. *JAMA Network Open*.

eReferences of included studies (n=133)

1. Huang K, Tao F, Faragher B, et al. A mixed-method study of factors associated with differences in caesarean section rates at community level: The case of rural China. Midwifery 2013;29(8):911-20. doi: 10.1016/j.midw.2012.11.003

2. Kale I. Does continuous cardiotocography during labor cause excessive fetal distress diagnosis and unnecessary cesarean sections? Journal of Maternal-Fetal and Neonatal Medicine 2021 doi: 10.1080/14767058.2021.1906220

3. Khresheh R, Barclay L. Knowledge, attitude and experience of episiotomy practice among obstetricians and midwives in Jordan. Women Birth 2020;33(2):e176-e81. doi: 10.1016/j.wombi.2019.03.007

4. Liao Z, Zhou Y, Li H, et al. The Rates and Medical Necessity of Cesarean Delivery in the Era of the Two-Child Policy in Hubei and Gansu Provinces, China. Am J Public Health 2019;109(3):476-82. doi: 10.2105/ajph.2018.304868

5. Long Q, Zhang Y, Zhang J, et al. Changes in caesarean section rates in China during the period of transition from the one-child to two-child policy era: cross-sectional National Household Health Services Surveys. BMJ Open 2022;12(4):e059208. doi: 10.1136/bmjopen-2021-059208

6. Mandong BM, Madaki AJ. Missed diagnosis of schistosomiasis leading to unnecessary surgical procedures in Jos University Teaching Hospital. Trop Doct 2005;35(2):96-7. doi: 10.1258/0049475054037011

7. Maurya PB, Bapsy PP, Chandrashekar M, et al. Surgical treatment of early breast cancer in a developing country. Annals of Oncology 2015;26:ix33. doi: 10.1093/annonc/mdv519.64

8. Wang E. Requests for cesarean deliveries: The politics of labor pain and pain relief in Shanghai, China. Soc Sci Med 2017;173:1-8. doi: 10.1016/j.socscimed.2016.11.032

9. Rao C, Zheng Z, Hu S, et al. The association of guideline adherence of coronary revascularization and clinical outcomes for patients with stable and complex coronary artery disease. Circulation 2015;132

10. Abdel-Aleem H, Darwish A, Abdelaleem AA, et al. Usefulness of the WHO C-Model to optimize the cesarean delivery rate in a tertiary hospital setting. Int J Gynaecol Obstet 2017;137(1):40-44. doi: 10.1002/ijgo.12092

11. Aminu M, Utz B, Halim A, et al. Reasons for performing a caesarean section in public hospitals in rural Bangladesh. BMC Pregnancy Childbirth 2014;14:130. doi: 10.1186/1471-2393-14-130

© 2023 Albarqouni L et al. *JAMA Network Open*.

12. Bakker W, van Dorp E, Kazembe M, et al. Management of prolonged first stage of labour in a low-resource setting: lessons learnt from rural Malawi. BMC Pregnancy Childbirth 2021;21(1):398. doi: 10.1186/s12884-021-03856-9

13. Beyene MG, Zemedu TG, Gebregiorgis AH, et al. Cesarean delivery rates, hospital readiness and quality of clinical management in Ethiopia: national results from two cross-sectional emergency obstetric and newborn care assessments. BMC Pregnancy Childbirth 2021;21(1):571. doi: 10.1186/s12884-021-04008-9

14. Deng R, Tang X, Liu J, et al. Cesarean delivery on maternal request and its influencing factors in Chongqing, China. BMC Pregnancy Childbirth 2021;21(1):384. doi: 10.1186/s12884-021-03866-7

15. Estellita Lins F, Fortney JA. Cesarean section in four Rio de Janeiro hospitals. International Journal of Gynecology and Obstetrics 1981;19(1):27-34. doi: 10.1016/0020-7292(81)90035-7

16. Feng XL, Wang Y, An L, et al. Cesarean section in the People's Republic of China: Current perspectives. International Journal of Women's Health 2014;6(1):59-74. doi: 10.2147/IJWH.S41410

17. França GV, Restrepo-Méndez MC, Maia MF, et al. Coverage and equity in reproductive and maternal health interventions in Brazil: impressive progress following the implementation of the Unified Health System. Int J Equity Health 2016;15(1):149. doi: 10.1186/s12939-016-0445-2

18. Gu N, Dai Y, Lu D, et al. Evaluation of cesarean delivery rates in different levels of hospitals in Jiangsu Province, China, using the 10-Group classification system. J Matern Fetal Neonatal Med 2021:1-7. doi: 10.1080/14767058.2021.1887124

19. Haider MR, Rahman MM, Moinuddin M, et al. Ever-increasing Caesarean section and its economic burden in Bangladesh. PLoS One 2018;13(12):e0208623. doi: 10.1371/journal.pone.0208623

20. Hassan EMA. Analysis of caesarean section rate according to the 10 group robson classification in zagazig university hospital. European Journal of Molecular and Clinical Medicine 2021;8(3):2708-15.

21. Howes R, Webster C, Garner J. Appendicitis in a deployed military setting: diagnosis, management and impact on the fighting force. J R Army Med Corps 2017;163(2):111-14. doi: 10.1136/jramc-2015-000614

22. Hoxha I, Fejza A, Aliu M, et al. Health system factors and caesarean sections in Kosovo: a cross-sectional study. BMJ Open 2019;9(4):e026702. doi: 10.1136/bmjopen-2018-026702

23. Kouanda S, Coulibaly A, Ouedraogo A, et al. Audit of cesarean delivery in Burkina Faso. Int J Gynaecol Obstet 2014;125(3):214-8. doi: 10.1016/j.ijgo.2013.11.010

24. Leone T. Demand and supply factors affecting the rising overmedicalization of birth in India. Int J Gynaecol Obstet 2014;127(2):157-62. doi: 10.1016/j.ijgo.2014.05.018

© 2023 Albarqouni L et al. *JAMA Network Open*.

25. Liang J, Mu Y, Li X, et al. Relaxation of the one child policy and trends in caesarean section rates and birth outcomes in China between 2012 and 2016: observational study of nearly seven million health facility births. Bmj 2018;360:k817. doi: 10.1136/bmj.k817

26. Long Q, Klemetti R, Wang Y, et al. High Caesarean section rate in rural China: is it related to health insurance (New Co-operative Medical Scheme)? Soc Sci Med 2012;75(4):733-7. doi: 10.1016/j.socscimed.2012.03.054

27. Majlesi M, Montazeri A, Rakhshani F, et al. 'No to unnecessary caesarean sections': Evaluation of a mass-media campaign on women's knowledge, attitude and intention for mode of delivery. PLoS One 2020;15(8):e0235688. doi: 10.1371/journal.pone.0235688

28. Mohammadi S, Fallahian M, Gargari SS, et al. Audits and the robson classification reveal maternal consequences of inappropriate cesareans. International Journal of Gynecology and Obstetrics 2018;143:643. doi: 10.1002/ijgo.12583

29. Nelson JP. Indications and appropriateness of caesarean sections performed in a tertiary referral centre in Uganda: a retrospective descriptive study. Pan Afr Med J 2017;26:64. doi: 10.11604/pamj.2017.26.64.9555

30. Elnakib S, Abdel-Tawab N, Orbay D, et al. Medical and non-medical reasons for cesarean section delivery in Egypt: a hospital-based retrospective study. BMC Pregnancy Childbirth 2019;19(1):411. doi: 10.1186/s12884-019-2558-2

31. Ganji F, Yusefi H, Baradaran A. Effect of a participatory intervention to reduce the number of unnecessary cesarean sections performed in Shahrekord of Iran. Journal of Medical Sciences 2006;6(4):690-92.

32. Gonzalez-Perez GJ, Vega-Lopez MG, Cabrera-Pivaral C, et al. Caesarean sections in Mexico: are there too many? Health Policy Plan 2001;16(1):62-7. doi: 10.1093/heapol/16.1.62

33. Hakimi S, Nikan F, Mahram BS, et al. Decreasing unnecessary cesarean section rate in north west iran: A story from achievements and challenges. Current Women's Health Reviews 2020;16(1):23-25. doi: 10.2174/1573404815666190823111132

34. Hatamleh R, Abujilban S, Al-Shraideh AJ, et al. Maternal request for cesarian birth without medical indication in a group of healthy women: A qualitative study in Jordan. Midwifery 2019;79:102543. doi: 10.1016/j.midw.2019.102543

35. Hoxha I, Fink G. Caesarean sections and health financing: a global analysis. BMJ Open 2021;11(5):e044383. doi: 10.1136/bmjopen-2020-044383

36. Hu Y, Tao H, Cheng Z. Caesarean Sections in Beijing, China - Results from a Descriptive Study. Gesundheitswesen 2016;78(1):e1-5. doi: 10.1055/s-0035-1549937

37. Ion RC, Allott H, Keightley A, et al. Improving decision-making in labour to reduce unnecessary caesarean sections in a rural hospital in south-west Uganda. BJOG: An International Journal of Obstetrics and Gynaecology 2013;120:330. doi: 10.1111/1471-0528.12297

38. Maaløe N, Bygbjerg IC, Onesmo R, et al. Disclosing doubtful indications for emergency cesarean sections in rural hospitals in Tanzania: a retrospective

© 2023 Albarqouni L et al. *JAMA Network Open*.

criterion-based audit. Acta Obstet Gynecol Scand 2012;91(9):1069-76. doi: 10.1111/j.1600-0412.2012.01474.x

39. Perrotta C, Romero M, Sguassero Y, et al. Caesarean birth in public maternities in Argentina: a formative research study on the views of obstetricians, midwives and trainees. BMJ Open 2022;12(1):e053419. doi: 10.1136/bmjopen-2021-053419

40. Schantz C, Aboubakar M, Traoré AB, et al. Caesarean section in Benin and Mali: increased recourse to technology due to suffering and under-resourced facilities. Reprod Biomed Soc Online 2020;10:10-18. doi: 10.1016/j.rbms.2019.12.001

41. Sriussadaporn S, Tanphiphat C, Poomsuwan P. Can unnecessary operations for abdominal stab would be safely reduced? A review of 255 patients. 1993

42. Su Y, Heitner J, Yuan C, et al. Effect of a Text Messaging-Based Educational Intervention on Cesarean Section Rates Among Pregnant Women in China: Quasirandomized Controlled Trial. JMIR Mhealth Uhealth 2020;8(11):e19953. doi: 10.2196/19953

43. Tang Y, Gao J, Sun L, et al. Promotion of Pre-natal Education Courses Is Associated With Reducing the Rates of Caesarean Section: A Case-Control Study. Front Public Health 2021;9:666337. doi: 10.3389/fpubh.2021.666337

44. Wang X, Hellerstein S, Hou L, et al. Caesarean deliveries in China. BMC Pregnancy Childbirth 2017;17(1):54. doi: 10.1186/s12884-017-1233-8

45. Yang J, Bai H. Knowledge, attitude and experience of episiotomy practice among obstetricians and midwives: a cross-sectional study from China. BMJ Open 2021;11(4):e043596. doi: 10.1136/bmjopen-2020-043596

46. Zarifsanaiey N, Bagheri A, Jahanpour F, et al. Effect of an Interactive Training on Choosing Delivery Method among Primiparous Pregnant Women: An Interventional Study. Invest Educ Enferm 2020;38(1) doi: 10.17533/udea.iee.v38n1e04

47. Kaboré C, Ridde V, Chaillet N, et al. DECIDE: a cluster-randomized controlled trial to reduce unnecessary caesarean deliveries in Burkina Faso. BMC Med 2019;17(1):87. doi: 10.1186/s12916-019-1320-y

48. Litorp H, Kidanto HL, Nystrom L, et al. Increasing caesarean section rates among low-risk groups: a panel study classifying deliveries according to Robson at a university hospital in Tanzania. BMC Pregnancy Childbirth 2013;13:107. doi: 10.1186/1471-2393-13-107

49. Muñoz-Enciso JM, Rosales-Aujang E, Domínguez-Ponce G, et al. [Cesarean birth: justifying indication or justified concern?]. Ginecol Obstet Mex 2011;79(2):67-74.

50. Peiris CJ. Inappropriate red cell transfusions. Vox Sanguinis 2010;99:424-25. doi: 10.1111/j.1423-0410.2010.01343-2.x

51. Smith-Oka V. Cutting Women: Unnecessary cesareans as iatrogenesis and obstetric violence. Social Science and Medicine 2022;296 doi: 10.1016/j.socscimed.2022.114734

52. Zarshenas M, Zhao Y, Binns CW, et al. Incidence and Determinants of Caesarean Section in Shiraz, Iran. Int J Environ Res Public Health 2020;17(16) doi:

© 2023 Albarqouni L et al. *JAMA Network Open*.

10.3390/ijerph17165632

53. Zhang L, Zhang L, Li M, et al. A cluster-randomized field trial to reduce cesarean section rates with a multifaceted intervention in Shanghai, China. BMC Med 2020;18(1):27. doi: 10.1186/s12916-020-1491-6

54. Ahmed HO, Muhedin R, Boujan A, et al. A five-year longitudinal observational study in morbidity and mortality of negative appendectomy in Sulaimani teaching Hospital/Kurdistan Region/Iraq. Sci Rep 2020;10(1):2028. doi: 10.1038/s41598-020-58847-1

55. Ahmed MS, Islam M, Jahan I, et al. Multilevel analysis to identify the factors associated with caesarean section in Bangladesh: evidence from a nationally representative survey. Int Health 2022 doi: 10.1093/inthealth/ihac006

56. da Matta Machado Fernandes L, Lansky S, Reis Passos H, et al. Brazilian women's use of evidence-based practices in childbirth after participating in the Senses of Birth intervention: A mixed-methods study. PLoS One 2021;16(4):e0248740. doi: 10.1371/journal.pone.0248740

57. Darsareh F, Aghamolaei T, Rajaei M, et al. B Butterfly Campaign: A social marketing campaign to promote normal childbirth among first-time pregnant women. Women Birth 2019;32(2):e166-e72. doi: 10.1016/j.wombi.2018.06.007

58. Desai S, Sinha T, Mahal A. Prevalence of hysterectomy among rural and urban women with and without health insurance in Gujarat, India. Reprod Health Matters 2011;19(37):42-51. doi: 10.1016/s0968-8080(11)37553-2

59. Dhakal Rai S, Regmi PR, Teijlingen EV, et al. Rising Rates of Caesarean Section in Urban Nepal. J Nepal Health Res Counc 2019;16(41):479-80.

60. Karthikeyan G, Shirodkar U, Lochan MR, et al. Appropriateness-based reimbursement of elective invasive coronary procedures in low- and middle-income countries: Preliminary assessment of feasibility in India. Natl Med J India 2017;30(1):11-14.

61. Khan RN, Baig M, Khan S, et al. Clinical relevance of axillary lymph node dissection in cytology proven lymph node positive axilla after neo adjuvant systemic therapy. European Journal of Surgical Oncology 2018;44(6):897-98.

62. Liabsuetrakul T, Sukmanee J, Thungthong J, et al. Trend of cesarean section rates and correlations with adverse maternal and neonatal outcomes: A secondary analysis of thai universal coverage scheme data. AJP Reports 2019;9(4):E328-E36. doi: 10.1055/s-0039-1697656

63. Litorp H, Mgaya A, Mbekenga CK, et al. Fear, blame and transparency: Obstetric caregivers' rationales for high caesarean section rates in a low-resource setting. Soc Sci Med 2015;143:232-40. doi: 10.1016/j.socscimed.2015.09.003

64. Belizán JM, Althabe F, Barros FC, et al. Rates and implications of caesarean sections in Latin America: ecological study. Bmj 1999;319(7222):1397-400. doi: 10.1136/bmj.319.7222.1397

65. Gedefaw G, Waltengus F, Demis A. Does Timing of Antenatal Care Initiation and the Contents of Care Have Effect on Caesarean Delivery in Ethiopia? Findings from Demographic and Health Survey. J Environ Public Health 2021;2021:7756185. doi: 10.1155/2021/7756185

© 2023 Albarqouni L et al. *JAMA Network Open*.

66. Giang HTN, Ulrich S, Tran HT, et al. Monitoring and interventions are needed to reduce the very high Caesarean section rates in Vietnam. Acta Paediatr 2018;107(12):2109-14. doi: 10.1111/apa.14376

67. Islam MT, Yoshimura Y. Rate of cesarean delivery at hospitals providing emergency obstetric care in Bangladesh. Int J Gynaecol Obstet 2015;128(1):40-3. doi: 10.1016/j.ijgo.2014.07.021

68. Kaboré C, Ridde V, Kouanda S, et al. Determinants of non-medically indicated cesarean deliveries in Burkina Faso. Int J Gynaecol Obstet 2016;135 Suppl 1:S58-s63. doi: 10.1016/j.ijgo.2016.08.019

69. Lin S, Zhang H, Rao CF, et al. Assessing the association of appropriateness of coronary revascularization and 1-year clinical outcomes for patients with stable coronary artery disease in China. Chin Med J (Engl) 2020;133(1):1-8. doi: 10.1097/cm9.0000000000000592

70. Mumtaz S, Bahk J, Khang YH. Rising trends and inequalities in cesarean section rates in Pakistan: Evidence from Pakistan Demographic and Health Surveys, 1990-2013. PLoS One 2017;12(10):e0186563. doi: 10.1371/journal.pone.0186563

71. Mumtaz Z, Bhatti A, Salway S. Challenges to achieving appropriate and equitable access to Caesarean section: ethnographic insights from rural Pakistan. J Biosoc Sci 2020;52(4):491-503. doi: 10.1017/s0021932019000567

72. Ostovar R, Pourreza A, Rashidian A, et al. Appropriateness of cesarean sections using the RAND Appropriateness Method criteria. Arch Iran Med 2012;15(1):8-13.

73. Patil D, Lanjewar C, Vaggar G, et al. Appropriateness of elective percutaneous coronary intervention and impact of government health insurance scheme - A tertiary centre experience from Western India. Indian Heart J 2017;69(5):600-06. doi: 10.1016/j.ihj.2016.12.018

74. Rafiq MS, Khan MM, Khan A, et al. Receiver operator characteristic curve analysis of the Lintula score for reduction of negative appendectomies in adults. J Coll Physicians Surg Pak 2015;25(2):100-3.

75. Santas G, Santas F. Trends of caesarean section rates in Turkey. J Obstet Gynaecol 2018;38(5):658-62. doi: 10.1080/01443615.2017.1400525

76. Srisomboon J, Pantusart A, Phongnarisorn C, et al. Reasons for improper simple hysterectomy in patients with invasive cervical cancer in the northern region of Thailand. J Obstet Gynaecol Res 2000;26(3):175-80. doi: 10.1111/j.1447-0756.2000.tb01307.x

77. van den Bergh JE, Sueters M, Segaar M, et al. Determinants of episiotomy in rural Zimbabwe. Acta Obstet Gynecol Scand 2003;82(10):966-8. doi: 10.1034/j.1600-0412.2003.00323.x

78. Zamboni K, Schellenberg J, Hanson C, et al. Assessing scalability of an intervention: why, how and who? Health Policy Plan 2019;34(7):544-52. doi: 10.1093/heapol/czz068

79. Ahmmed F, Manik MMR, Hossain MJ. Caesarian section (CS) delivery in Bangladesh: A nationally representative cross-sectional study. PLoS One

© 2023 Albarqouni L et al. *JAMA Network Open*.

2021;16(7):e0254777. doi: 10.1371/journal.pone.0254777

80. Al Rifai R. Rising cesarean deliveries among apparently low-risk mothers at university teaching hospitals in Jordan: analysis of population survey data, 2002-2012. Glob Health Sci Pract 2014;2(2):195-209. doi: 10.9745/ghsp-d-14-00027

81. Boatin AA, Schlotheuber A, Betran AP, et al. Within country inequalities in cesarean section rates: Observational study of 72 low and middle income countries. Obstetrical and Gynecological Survey 2018;73(6):333--34. doi: 10.1097/OGX.0000000000000573

82. Cheng K, Yuan M, Xu C, et al. A chest tube may not necessary in children thoracoscopic lobectomy. Medicine (Baltimore) 2019;98(26):e15857. doi: 10.1097/md.0000000000015857

83. Phadungkiatwattana P, Tongsakul N. Analyzing the impact of private service on the cesarean section rate in public hospital Thailand. Arch Gynecol Obstet 2011;284(6):1375-9. doi: 10.1007/s00404-011-1867-0

84. Ribeiro LB. Nascer em Belo Horizonte: cesarianas desnecessárias e prematuridade, 2016:117-17.

85. Rudey EL, Leal MDC, Rego G. Cesarean section rates in Brazil: Trend analysis using the Robson classification system. Medicine (Baltimore) 2020;99(17):e19880. doi: 10.1097/md.0000000000019880

86. Saleh Gargari S, Essén B, Fallahian M, et al. Auditing the appropriateness of cesarean delivery using the Robson classification among women experiencing a maternal near miss. Int J Gynaecol Obstet 2019;144(1):49-55. doi: 10.1002/ijgo.12698

87. Shirzad M, Shakibazadeh E, Hajimiri K, et al. Prevalence of and reasons for women's, family members', and health professionals' preferences for cesarean section in Iran: a mixed-methods systematic review. Reprod Health 2021;18(1):3. doi: 10.1186/s12978-020-01047-x

88. Singh P, Hashmi G, Swain PK. High prevalence of cesarean section births in private sector health facilities- analysis of district level household survey-4 (DLHS-4) of India. BMC Public Health 2018;18(1):613. doi: 10.1186/s12889-018-5533-3

89. Torloni MR, Brizuela V, Betran AP. Mass media campaigns to reduce unnecessary caesarean sections: a systematic review. BMJ Glob Health 2020;5(2):e001935. doi: 10.1136/bmjgh-2019-001935

90. Türkmen A, Temel M. Algorithmic approach to the prevention of unnecessary fasciotomy in extremity snake bite. Injury 2016;47(12):2822-27. doi: 10.1016/j.injury.2016.10.023

91. Takegata M, Smith C, Nguyen HAT, et al. Reasons for Increased Caesarean Section Rate in Vietnam: A Qualitative Study among Vietnamese Mothers and Health Care Professionals. Healthcare (Basel) 2020;8(1) doi: 10.3390/healthcare8010041

92. Tenge RK, Ndungu JM. Blunt abdominal trauma in children at Kenyatta National Hospital. East Afr Med J 1999;76(10):580-2.

© 2023 Albarqouni L et al. *JAMA Network Open*.

93. Ostovar R, Rashidian A, Pourreza A, et al. Developing criteria for cesarean section using the RAND appropriateness method. BMC Pregnancy Childbirth 2010;10:52. doi: 10.1186/1471-2393-10-52

94. Rahman MM, Haider MR, Moinuddin M, et al. Determinants of caesarean section in Bangladesh: Cross-sectional analysis of Bangladesh Demographic and Health Survey 2014 Data. PLoS One 2018;13(9):e0202879. doi: 10.1371/journal.pone.0202879

95. Rubagumya F, Mitera G, Ka S, et al. Choosing Wisely Africa: Ten Low-Value or Harmful Practices That Should Be Avoided in Cancer Care. JCO Glob Oncol 2020;6:1192-99. doi: 10.1200/go.20.00255

96. Colomar M, Cafferata ML, Aleman A, et al. Mode of childbirth in low-risk pregnancies: Nicaraguan physicians' viewpoints. Matern Child Health J 2014;18(10):2382-92. doi: 10.1007/s10995-014-1478-z

97. Colomar M, Opiyo N, Kingdon C, et al. Do women prefer caesarean sections? A qualitative evidence synthesis of their views and experiences. PLoS One 2021;16(5):e0251072. doi: 10.1371/journal.pone.0251072

98. Dankwah E, Kirychuk S, Zeng W, et al. Socioeconomic inequalities in the use of caesarean section delivery in Ghana: a cross-sectional study using nationally representative data. Int J Equity Health 2019;18(1):162. doi: 10.1186/s12939-019-1063-6

99. Fernandes LMM, Lansky S, Oliveira BJ, et al. Changes in perceived knowledge about childbirth among pregnant women participating in the Senses of Birth intervention in Brazil: a cross-sectional study. BMC Pregnancy Childbirth 2020;20(1):265. doi: 10.1186/s12884-020-02874-3

100. Ghotbi Fa. Women's knowledge and attitude towards mode of delivery and frequency of cesarean section on mother's request in six public and private hospitals in Tehran, Iran, 2012. J Obstet Gynaecol Res 2014;40(5):1257--66. doi: 10.1111/jog.12335

101. Ola ER, Bello O, Abudu OO, et al. Episiotomies in Nigeria--should their use be restricted? Niger Postgrad Med J 2002;9(1):13-6.

102. Shi Y, Jiang Y, Zeng Q, et al. Influencing factors associated with the mode of birth among childbearing women in Hunan Province: a cross-sectional study in China. BMC Pregnancy Childbirth 2016;16:108. doi: 10.1186/s12884-016-0897-9 [published Online First: 20160516]

103. Wang L, Xu X, Baker P, et al. Factors associated with intention to have caesarean delivery in pregnant women in China: a cross-sectional analysis. The Lancet 2016;388:S2. doi: https://doi.org/10.1016/S0140-6736(16)31929-8

104. Zimmo K, Laine K, Fosse E, et al. Episiotomy practice in six Palestinian hospitals: a population-based cohort study among singleton vaginal births. BMJ Open 2018;8(7):e021629. doi: 10.1136/bmjopen-2018-021629

105. Prado DS, Mendes RB, Gurgel RQ, et al. Practices and obstetric interventions in women from a state in the Northeast of Brazil. Rev Assoc Med Bras (1992) 2017;63(12):1039-48. doi: 10.1590/1806-9282.63.12.1039

106. Roy N, Mishra PK, Mishra VK, et al. Changing scenario of C-section delivery in India: Understanding the maternal health concern and its associated

© 2023 Albarqouni L et al. *JAMA Network Open*.

predictors. J Family Med Prim Care 2021;10(11):4182-88. doi: 10.4103/jfmpc.jfmpc_585_21

107. Schantz C, Ravit M, Traoré AB, et al. Why are caesarean section rates so high in facilities in Mali and Benin? Sex Reprod Healthc 2018;16:10-14. doi: 10.1016/j.srhc.2018.01.001

108. Shirzad M, Shakibazadeh E, Betran AP, et al. Women's perspectives on health facility and system levels factors influencing mode of delivery in Tehran: a qualitative study. Reprod Health 2019;16(1):15. doi: 10.1186/s12978-019-0680-2

109. Suwanrath C, Chunuan S, Matemanosak P, et al. Why do pregnant women prefer cesarean birth? A qualitative study in a tertiary care center in Southern Thailand. BMC Pregnancy Childbirth 2021;21(1):23. doi: 10.1186/s12884-020-03525-3

110. Taviloglu K, Günay K, Ertekin C, et al. Abdominal stab wounds: the role of selective management. Eur J Surg 1998;164(1):17-21. doi: 10.1080/110241598750004904

111. Dias MA, Domingues RM, Schilithz AO, et al. Factors associated with cesarean delivery during labor in primiparous women assisted in the Brazilian Public Health System: data from a National Survey. Reprod Health 2016;13(Suppl 3):114. doi: 10.1186/s12978-016-0231-z

112. Galvao R, Hawley NL, da Silva CS, et al. How obstetricians and pregnant women decide mode of birth in light of a recent regulation in Brazil. Women Birth 2018;31(5):e310--e17. doi: 10.1016/j.wombi.2017.11.011

113. Gmez OL, Carrasquilla G. Factors associated with unjustified Cesarean section in four hospitals in Cali, Colombia. Int J Qual Health Care 1999;11(5):385--89. doi: 10.1093/intqhc/11.5.385

114. Kunthonkitidej K, Ngernset O. Self-evaluation of obstetricians by delivery data to reduce cesarean section rate in Chai Nat Hospital. J Med Assoc Thai 2001;84(11):1587--93.

115. Lansky S, Oliveira B, Ventura de-Souza Ka. Senses of birth-education and mobilization to decrease cesarean-section and Prematurity in Brazil. International Journal of Gynecology and Obstetrics 2018;143:500--01. doi: 10.1002/ijgo.12582

116. Lukas E, Enke G, Florian B, et al. Infantile Hepatic Hemangioma: Avoiding Unnecessary Invasive Procedures. Pediatric Gastroenterology, Hepatology & Nutrition 2020:72--78.

117. Misaeli C, Mgaya A, Kamala B, et al. Factors associated with women's intention of requesting caesarean delivery in Dar es Salaam, Tanzania, 2017:126.

118. Dekker L, Houtzager T, Kilume O, et al. Caesarean section audit to improve quality of care in a rural referral hospital in Tanzania. BMC Pregnancy Childbirth 2018;18(1):164. doi: 10.1186/s12884-018-1814-1

119. Ajeet S, Nandkishore K. The boom in unnecessary caesarean surgeries is jeopardizing women's health. Health Care Women Int 2013;34(6):513-21. doi: 10.1080/07399332.2012.721416

© 2023 Albarqouni L et al. *JAMA Network Open*.

120. Ahmed MS, Khan S, Yunus FM. Factors associated with the utilization of reproductive health services among the Bangladeshi married women: Analysis of national representative MICS 2019 data. Midwifery 2021;103:103139. doi: 10.1016/j.midw.2021.103139 [published Online First: 20210914]

121. Albertal M, Candiello A, Cura FA, et al. Evaluación de las revascularizaciones coronarias denominadas inapropiadas por la nueva clasificación de adecuación de procedimientos en un centro cardiovascular de alta complejidad. Rev argent cardiol 2010;78(6):507-11.

122. Alcantara LLM, Almeida NKO, Almeida R. Pattern of Live Births in Rio de Janeiro State, Brazil, According to Robson Groups and the Kotelchuck Index Classification - 2015/2016. Rev Bras Ginecol Obstet 2020;42(7):373-79. doi: 10.1055/s-0040-1712122

123. Al Rifai RH. Trend of caesarean deliveries in Egypt and its associated factors: evidence from national surveys, 2005-2014. BMC Pregnancy Childbirth 2017;17(1):417. doi: 10.1186/s12884-017-1591-2 [published Online First: 20171213]

124. Ali Y, Khan MW, Mumtaz U, et al. Identification of factors influencing the rise of cesarean sections rates in Pakistan, using MCDM. Int J Health Care Qual Assur 2018;31(8):1058-69. doi: 10.1108/ijhcqa-04-2018-0087

125. Althabe F, Belizán JM, Bergel E. Episiotomy rates in primiparous women in Latin America: hospital based descriptive study. Bmj 2002;324(7343):945-6. doi: 10.1136/bmj.324.7343.945

126. Althabe F, Belizán JM, Villar J, et al. Mandatory second opinion to reduce rates of unnecessary caesarean sections in Latin America: a cluster randomised controlled trial. Lancet 2004;363(9425):1934-40. doi: 10.1016/s0140-6736(04)16406-4

127. Alzate MM, Dongarwar D, Matas JL, et al. Phenotypes and markers of cesarean delivery among Colombian women. Int J Gynaecol Obstet 2019;147(2):187-94. doi: 10.1002/ijgo.12942

128. Bakker W, Bakker E, Huigens C, et al. Impact of Medical Doctors Global Health and Tropical Medicine on decision-making in caesarean section: a pre- and post-implementation study in a rural hospital in Malawi. Hum Resour Health 2020;18(1):87. doi: 10.1186/s12960-020-00516-5

129. Betran AP, Ye J, Moller AB, et al. Trends and projections of caesarean section rates: global and regional estimates. BMJ Glob Health 2021;6(6) doi: 10.1136/bmjgh-2021-005671

130. Bhatia M, Dwivedi L, Banerjee K, et al. An epidemic of avoidable caesarean deliveries in the private sector in India: Is physician-induced demand at play? Social Science & Medicine Vol 265 2020, ArtID 113511 2020;265 doi: https://dx.doi.org/10.1016/j.socscimed.2020.113511

131. Campero L, Hernández B, Leyva A, et al. [Trends in caesarean sections associated with non-clinical factors in a Birthing Educational Center in Mexico City]. Salud Publica Mex 2007;49(2):118-25. doi: 10.1590/s0036-36342007000200007

132. Chanrachakul B, Herabutya Y, Udomsubpayakul U. Epidemic of cesarean section at the general, private and university hospitals in Thailand. J Obstet Gynaecol Res 2000;26(5):357-61. doi: 10.1111/j.1447-0756.2000.tb01339.x

© 2023 Albarqouni L et al. *JAMA Network Open*.

133. Cheng YM, Yuan W, Cai WD, et al. [Study on the occurrence of cesarean section (CS) and factors related to CS in China]. Zhonghua Liu Xing Bing Xue Za Zhi 2003;24(10):893-6.

© 2023 Albarqouni L et al. *JAMA Network Open*.
