# Supplementary material for: Low-Value Surgical Procedures in Low- and Middle-Income Countries: A Systematic Scoping Review
Source: JAMA Netw Open. 2023 Nov 7;6(11):e2342215. doi: 10.1001/jamanetworkopen.2023.42215 (PMC10630901; doi:10.1001/jamanetworkopen.2023.42215)
Supplement: Supplement 2. — Data Sharing Statement [file jamanetwopen-e2342215-s002.pdf]

Data Sharing Statement

Albarqouni. Low-Value Surgical Procedures in Low- and Middle-Income Countries. *JAMA Netw Open*. Published November 08, 2023. doi:10.1001/jamanetworkopen.2023.42215

Data
Data available: Yes
Data types: Other (please specify)
Additional Information: Extracted data from included studies
How to access data: Contacting the corresponding author
When available: With publication

Supporting Documents
Document types: None

Additional Information
Who can access the data: Not applicable
Types of analyses: Not applicable
Mechanisms of data availability: Not applicable
